# Resensitizing multidrug-resistant Gram-negative bacteria to carbapenems and colistin using disulfiram

**DOI:** 10.1038/s42003-023-05173-7

**Published:** 2023-08-03

**Authors:** Chen Chen, Jinju Cai, Jingru Shi, Zhiqiang Wang, Yuan Liu

**Affiliations:** 1 Jiangsu Co-innovation Center for Prevention and Control of Important Animal Infectious Diseases and Zoonoses, College of Veterinary Medicine, Yangzhou University, Yangzhou, 225009 China; 2 Joint International Research Laboratory of Agriculture and Agri-Product Safety, The Ministry of Education of China, Yangzhou University, Yangzhou, 225009 China; 3 Institute of Comparative Medicine, Yangzhou University, Yangzhou, 225009 China

**Keywords:** Microbiology, Drug discovery

## Abstract

The increasing incidence of bacterial infections caused by multidrug-resistant (MDR) Gram-negative bacteria has deepened the need for new effective treatments. Antibiotic adjuvant strategy is a more effective and economical approach to expand the lifespan of currently used antibiotics. Herein, we uncover that alcohol-abuse drug disulfiram (DSF) and derivatives thereof are potent antibiotic adjuvants, which dramatically potentiate the antibacterial activity of carbapenems and colistin against New Delhi metallo-β-lactamase (NDM)- and mobilized colistin resistance (MCR)-expressing Gram-negative pathogens, respectively. Mechanistic studies indicate that DSF improves meropenem efficacy by specifically inhibiting NDM activity. Moreover, the robust potentiation of DSF to colistin is due to its ability to exacerbate the membrane-damaging effects of colistin and disrupt bacterial metabolism. Notably, the passage and conjugation assays reveal that DSF minimizes the evolution and spread of meropenem and colistin resistance in clinical pathogens. Finally, their synergistic efficacy in animal models was evaluated and DSF-colistin/meropenem combination could effectively treat MDR bacterial infections in vivo. Taken together, our works demonstrate that DSF and its derivatives are versatile and potent colistin and carbapenems adjuvants, opening a new horizon for the treatment of difficult-to-treat infections.

## Abstract (web summary)

Disulfiram and derivatives are adjuvants that aid treatment against antibiotic-resistant gram-negative bacteria by inhibiting New Delhi metallo-β-lactamase and enhancing membrane-damaging effects of antibiotics.

## Introduction

The emerging plasmid-borne resistance determinants have severely challenged the clinical efficacy of antibiotics, posing a global threat to public health system^1,2^. To combat bacterial infections caused by multidrug-resistant (MDR) pathogens, carbapenems and colistin have therefore been viewed as the last-resort antibiotics^3,4^. However, the discovery and spread of New Delhi metallo-β-lactamase 1 (NDM-1) in 2009 and mobilized colistin resistance gene (*mcr-1*)-encoding phosphoethanolamine transferases since 2015 substantially impaired the efficacy of meropenem and colistin, respectively^5,6^. Moreover, nearly all available β-lactam antibiotics lose effectiveness due to NDM-1 and other metallo-β-lactamases (MBLs). Meanwhile, the plasmid-borne mobile colistin resistance gene *mcr-1* and its variants, *mcr-2* to *mcr-10*, have already disseminated over 40 countries/regions and caused a transferable mechanism of colistin resistance^7^. Furthermore, the discovery and development of novel efficient antibiotics have stalled, exacerbating the drug resistance crisis^8^. To maximize antibiotic effectiveness while minimizing its side effects and the development of resistance, combination therapy has been recommended as a safer and more economic alternative^9^. For example, colistin showed time-dependent synergistic activity against multidrug-resistant (MDR) *Acinetobacter baumannii* in combination with aztreonam^10^, and synergistic killing of MDR Gram-negative pathogens with rifampicin^11–13^. Nevertheless, the combination of antibiotics with other antibiotics may lead to the emergence of MDR bacterial strains. By contrast, the combination of antibiotic and non-antibiotic compounds, generally also termed antibiotic adjuvants^14^, can avoid this defect while resensitizing drug-resistant bacteria to clinically important antibiotics^9^.

Disulfiram (DSF), an oral prescription drug, is known as an anti-alcoholism drug for the treatment of chronic alcoholism^15^. According to pharmacokinetic studies, the byproducts of DSF metabolism, such as diethyldithiocarbamate (DDTC) and diethylamine, accumulate toxic acetaldehyde during the conversion of ethanol to acetic acid and function as aldehyde dehydrogenase inhibitors^16^. In addition, other pharmacological functions of DSF, such as the prophylactic and therapeutic effects on diet-induced obesity^17^ and tumor-suppressing effects^18^, have also been partly demonstrated. Furthermore, several studies have investigated the direct antibacterial activity of DSF and its metabolites^16^. For instance, a previous study showed that DSF had equipotent antibacterial activity against *Staphylococcus aureus* with minimum inhibitory concentrations (MICs) of 8–16 mg/L, and eradicated staphylococcal biofilms as well as intracellular *S. aureus*^19^. DSF was also found to prevent in vitro growth of the oomycete *Pythium insidiosum* by inhibiting bacterial urease and aldehyde dehydrogenase activity^20^. Nevertheless, the potentiation of DSF and its metabolites to the existing antibiotics, particularly for carbapenems and colistin, is still poorly understood.

In this study, we comprehensively evaluated the potency of DSF and its derivatives as potential antibiotic adjuvants to restore the efficacy of clinically relevant antibiotics. Herein, we discovered that DSF and its derivatives effectively enhance the efficacy of meropenem and colistin both in vivo and in vitro, while drastically inhibiting the evolution and transmission of drug resistance. The discovery of DSF and derivatives thereof as potent antibiotic enhancers provides a promising combination strategy for fighting against carbapenem-resistant Enterobacterales and colistin-resistant Gram-negative pathogens.

## Results

### DSF and derivatives thereof are potent antibiotic adjuvants against MDR bacteria

**Fig. 1:**
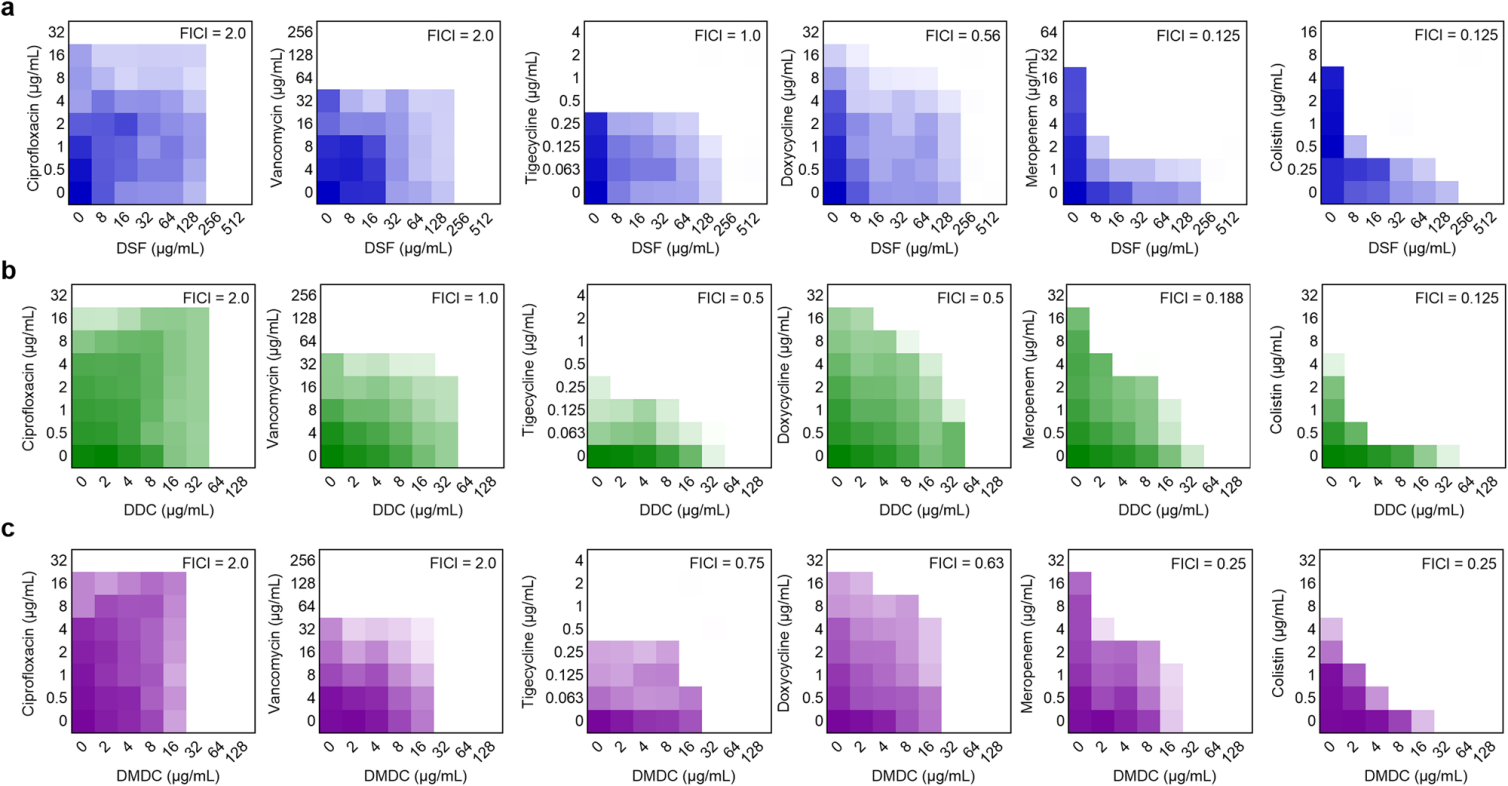
Checkerboard assays of DSF and its derivatives and various antibiotics against multidrug-resistant *E. coli* B2, related to Supplementary Table 2 and 3. The horizontal axis represents the concentrations of DSF (**a**), DDC (**b**) and DMDC (**c**), and the vertical axis represents the concentrations of six antibiotics. Darker color regions indicate higher cell density and lighter regions indicate lower cell density. Data represent the mean absorbance at 600 nm of biological replicates.

To investigate the potentiation of DSF and its derivatives (Supplementary Fig. 1) to the existing antibiotics, we performed checkerboard assays to evaluate their synergistic effects on multiple classes of antibiotics, including ciprofloxacin, vancomycin, tigecycline, doxycycline, meropenem and colistin, against an MDR isolate *E. coli* B2^21^, which confers resistance to almost all major classes of antibiotics. The fractional inhibitory concentration index (FICI) value was used to evaluate the drug interaction of two compounds (A and B) and calculated as the sum of FICI_A_ and FICI_B_. Notably, a comparison among these antibiotics revealed that the activity of meropenem and colistin was markedly enhanced in the presence of DSF, with a FICI of 0.125 for both (Fig. 1a), indicating that DSF had a synergistic antibacterial effect with meropenem and colistin. The relative MICs of meropenem and colistin were decreased from 32 to 2 µg/mL (16-fold) and from 8 to 0.25 µg/mL (32-fold) under one-quarter of the MIC of DSF (Supplementary Table 2). Additionally, the DSF derivatives, including diethyldithiocarbamate (DDC) and dimethyldithiocarbamate (DMDC), also showed potent synergistic activity with meropenem and colistin of all the antibiotics tested. Sub-MIC of DDC (16 µg/mL) substantially reduced their MICs by 16-fold while reversing meropenem and colistin resistance in *E. coli* B2 (FICI = 0.188/0.125, respectively) (Fig. 1b). Similarly, the MICs of meropenem and colistin against *E. coli* B2 were decreased by 8- and 16-fold when treated with sub-MIC of DMDC (8 µg/mL) (FICI = 0.25/0.25, respectively) (Fig. 1c and Supplementary Table 3). To validate the potentiation, we further evaluated these combinations on several types of Gram-negative bacteria, including drug-sensitive strains, MCR-producing Enterobacterales and MBL-positive Enterobacteriaceae. As demonstrated in Supplementary Table 4, DSF, DDC and DMDC fully restored the susceptibility of NDM-positive Gram-negative pathogens, but not NDM-negative bacteria. These NDM-positive Gram-negative pathogens carry *bla*_NDM-1_ or *bla*_NDM-5_ gene and are meropenem-resistant with MICs of 16-32 µg/mL. Additionally, we found that DSF and its metabolites (¼ MIC) effectively enhanced colistin activity against both MCR-negative and -positive Gram-negative pathogens by 4-64-fold (Supplementary Fig. 2, Supplementary Table 5), including colistin-susceptible bacteria and colistin-resistant *E. coli* DH5α (PUC19-*mcr-1*), *E. coli* G92 (*mcr-1*), *E. coli* CP131 (*mcr-3*) and *K. pneumoniae* D120 (*mcr-8*). It is worth noting that the colistin-resistant bacteria were 2-16-fold more potentiated than sensitive bacteria by DSF, DDC and DMDC in combination with colistin, respectively. These findings demonstrate that DSF and metabolites thereof are potent carbapenems and colistin adjuvants.

### DSF potentiates meropenem efficacy by specifically inhibiting NDM activity

**Fig. 2:**
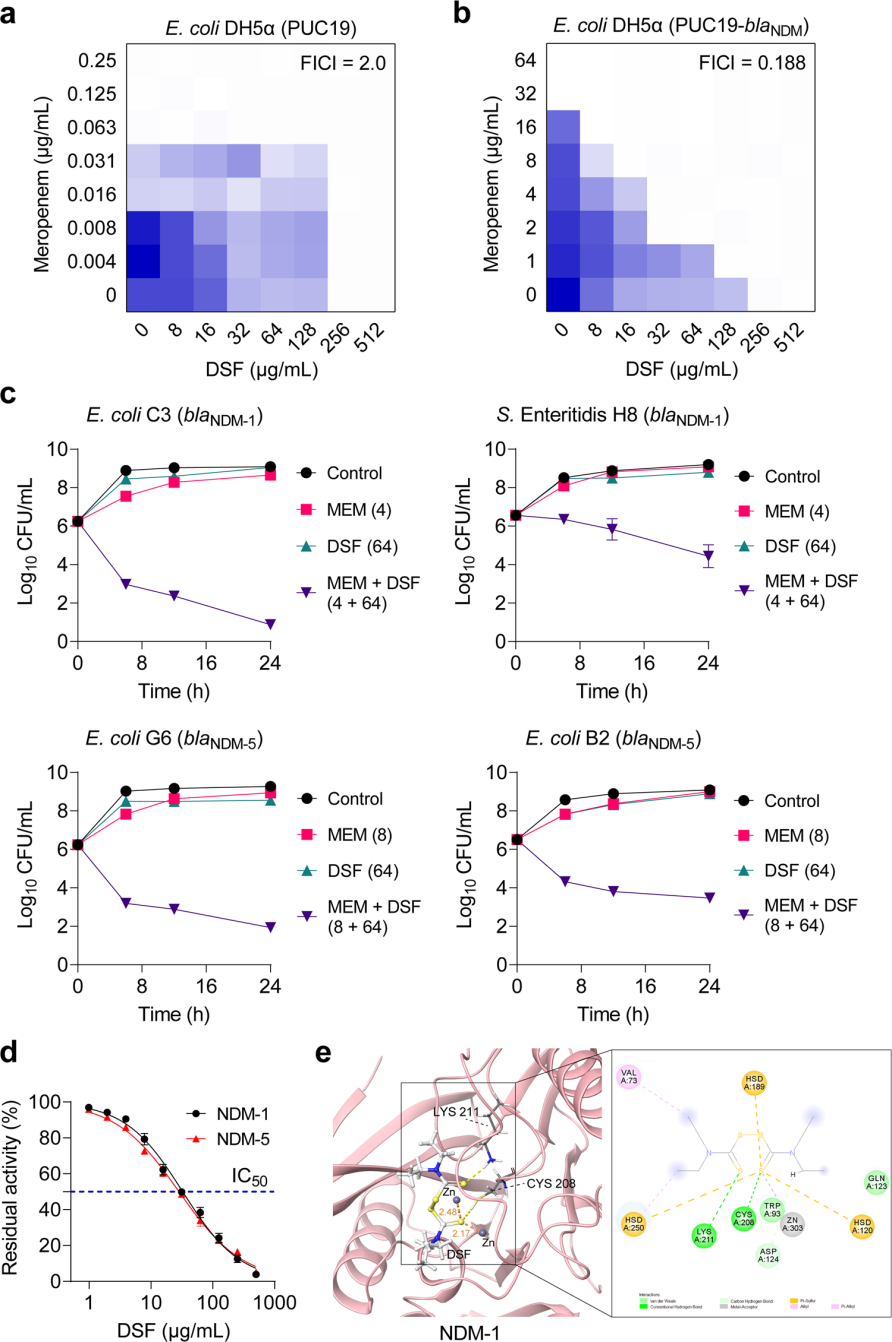
DSF restores the susceptibility of NDM-positive bacteria to meropenem. **a**, **b** Checkerboard assays between DSF and meropenem against *E. coli* DH5a (PUC19) (**a**) and *E. coli* DH5a (PUC19-*bla*_NDM-1_) (**b**). OD_600_ was measured after 18 h incubation at 37 °C. Data represent the mean OD_600_ of biological replicates. **c** Time-dependent killing curves under the combination treatment of meropenem and DSF against *E. coli* C3 (*bla*_NDM-1_), *S. enteritidis* H8 (*bla*_NDM-1_), *E. coli* G6 (*bla*_NDM-5_), and MDR *E. coli* B2 (*bla*_NDM-5_). Bacterial strains were grown to 4 h in MHB broth, then treated with PBS, meropenem (sub-MIC) or DSF (64 μg/mL) alone or in combination. The bacterial CFUs per mL at different time points during 24 h were determined. **d** Dose-dependent in vitro inhibitory effect of DSF on NDM-1 and NDM-5 activity. IC_50_, half-maximal inhibitory concentration. Data in (**c**) and (**d**) from three biological replicates were presented as mean ± SD. **e** Molecular docking analysis of the complexes of NDM-1 with DSF. In the close-up 2D view, the hydrogen bonds formed between them were depicted as green dotted line, and the residues involved in the hydrogen bond formation are CYS208 and LYS211, where one sulfur atom of DSF formed in coordination with Zn.

To further examine the synergistic effect of DSF with meropenem, checkerboard broth microdilution assay and time-dependent killing were further performed. Consistent with the above results, DSF remarkably enhanced meropenem activity against *E. coli* DH5α (PUC19-*bla*_NDM_) (FICI = 0.188) rather than *E. coli* DH5α (PUC19) (FICI = 2.0) (Fig. 2a, b), indicating that the action of DSF was related to the inhibition of resistance determinants. In addition, the time-kill dynamics were conducted on various NDM-positive pathogens to investigate the synergistic bactericidal effect of meropenem plus DSF. As shown in Fig. 2c, the combination of the sub-MICs of meropenem and DSF led to more than 4-log_10_ CFUs reduction of four tested NDM-positive bacteria during 24 h. By contrast, the identical concentrations of meropenem (4 or 8 µg/mL) and DSF (64 µg/mL) alone failed to prevent bacterial proliferation.

Considering the specific potentiating effect of DSF on NDM-positive bacteria, we hypothesized that DSF restores the susceptibility of drug-resistant bacteria to meropenem by targeting NDM enzyme. To test this, we assessed the enzymatic activity of NDM-1 and NDM-5 after exposure to increasing concentrations of DSF. As expected, DSF dose-dependently suppressed the activity of NDM-1 and NDM-5, with IC_50_ of 32.49 µg/mL and 28.32 µg/mL, respectively (Fig. 2d), suggesting that DSF can act as a novel NDM inhibitor. Next, molecular docking analysis was performed to explore how DSF interacts with NDM protein to inhibit its activity. Computer-simulated docking, using a grid box surrounding the active pocket structure of NDM-1 (PDB ID: 4EYL), indicated that DSF could fit into the active site and bind with CYS208 and LYS211 (Fig. 2e), with the lowest binding energy of −5.14 kcal/mol (Supplementary Table 7). Furthermore, the docking analysis of the complexes of NDM-1 with DDC and DMDC demonstrated that the DSF derivatives could also bind with the active pocket directly. As shown in Supplementary Fig. 6, DDC and DMDC could also bind with LYS211, which is important to NDM-1 activity^22–24^, with the lowest binding energy of −3.40 kcal/mol and −3.63 kcal/mol, respectively. Together, our results indicate that the underlying mechanism by which DSF and its derivatives resensitize NDM-positive bacteria to meropenem is attributable to their ability to bind and inhibit the activity of NDM.

### Disulfiram restores colistin activity against MCR-positive pathogens

**Fig. 3:**
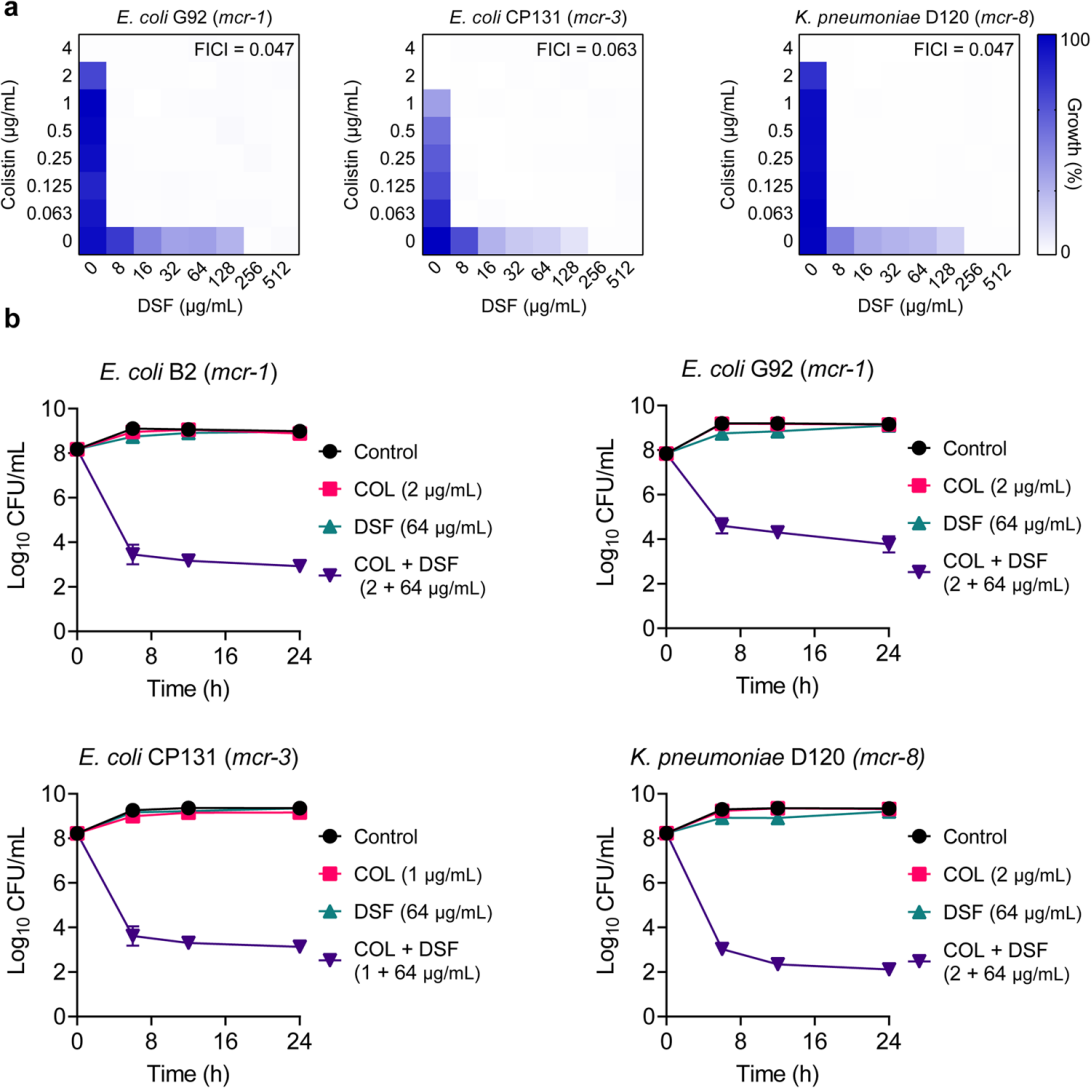
Potent synergistic activity of colistin and DSF against MCR-positive Gram-negative bacteria. **a** Checkerboard assays of DSF and colistin against MCR*-*positive Gram-negative bacteria, related to Supplementary Table 5. Dark blue regions represent higher cell density. Data represent the mean OD value at 600 nm of biological replicates. **b** Time-dependent killing curves of *mcr*-positive bacteria by the combination treatment of colistin and DSF. MDR *E. coli* B2 (*mcr-1*), *E. coli* G92 (*mcr-1*), *E. coli* CP131 (*mcr-3*), and *K. pneumoniae* D120 (*mcr-8*) were grown to 4 h in MHB broth, then treated with PBS, colistin (sub-MIC) or DSF (64 μg/mL) alone or in combination. The bacterial CFUs per mL at different time points during 24 h were determined. Data from three biological replicates were presented as mean ± SD.

To further assess the synergistic activity between DSF and colistin, checkerboard assays were also performed in three clinical MCR-positive isolates, including *E. coli* G92 (*mcr-1*), *E. coli* CP131 (*mcr-3*) and *K. pneumonine* D120 (*mcr-8*). Surprisingly, the combination of DSF and colistin displayed unprecedented synergistic antibacterial activity against these *mcr*-carrying pathogens, with FICI values of 0.047, 0.063, and 0.047, respectively (Fig. 3a). To explore the synergistic bactericidal activity of this combination, time-dependent killing curves for different MCR-positive Gram-negative bacteria were also determined. The results revealed that all colistin-resistant bacteria were effectively eliminated in a time-dependent manner under DSF-colistin combination treatment (Fig. 3b), whereas the corresponding concentrations of the two compounds alone have no effect on bacterial growth.

Considering the formation of biofilms dramatically affects the efficacy of antibiotics, formation and eradication of biofilms experiments were performed to explore the influence of DSF. Bacterial biofilms were cultured in the presence of different concentrations of colistin alone or in combination with DSF at 64 μg/mL. As shown in Supplementary Fig. 3a, the supplementary of DSF extensively prevented the formation of biofilms compared with colistin alone. Furthermore, DSF in combination with colistin displayed a significant eradicable effect on mature biofilm in a dose-dependent manner (Supplementary Fig. 3b). Taken together, these data indicate that DSF greatly potentiates colistin activity against MCR-positive pathogens in different metabolic states.

### DSF enhances membrane damage ability of colistin and disrupts bacterial metabolism

**Fig. 4:**
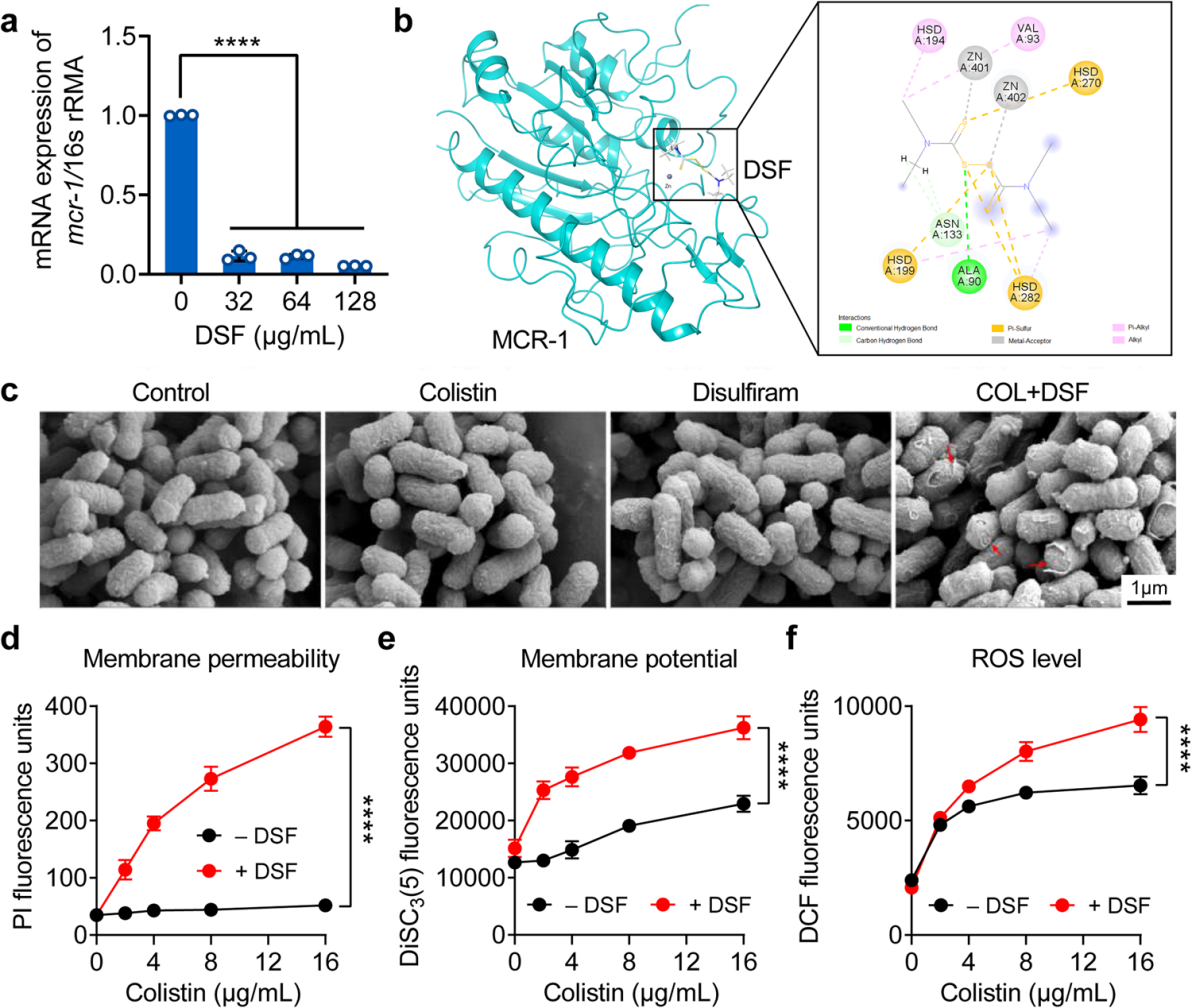
DSF exacerbates membrane damage and oxidative damage of colistin. **a** mRNA expression of *mcr-1* gene after exposure to increasing concentrations of DSF. Data analysis was performed using 2^-(∆∆Ct) method, with 16S rRNA serving as the housekeeping gene. Data were presented as mean ± SD of three biological replicates and statistical significance was analyzed by one-way ANOVA (****P < 0.0001). **b** Molecular docking analysis of the complexes of MCR-1 protein with DSF. The compound is involved in the metal coordination formation with the active-site zinc ion, marked by gray lines. **c** Scanning electron microscopy (SEM) images of *E. coli* B2 incubated with colistin, DSF and the combination of them. Scar bar, 1 μm. **d**–**f** Outer membrane permeability (**d**), membrane potential (**e**) and ROS level (**f**) of *E. coli* B2 under different concentrations of colistin with or without DSF (64 μg/mL), determined by monitoring the fluorescence intensity of propidium iodide (PI), 3,3’-dipropylthiadicarbocyanine iodide (DiSC_3_(5)) and 2’,7’-dichlorodihydrofluorescein diacetate (DCFH-DA), respectively. Data were expressed as mean ± SD of three biological replicates and statistical significance was analyzed by two-way ANOVA with Sidak’s multiple comparisons test (****P < 0.0001).

The above results revealed that the potentiating activity of DSF to colistin maybe related to the resistance determinant *mcr*, thus RT-qPCR analysis was further conducted to evaluate the expression of *mcr-1* gene in *E. coli* B2 exposed to sub-MICs of DSF. As demonstrated in Fig. 4a, DSF significantly inhibited mRNA expression of *mcr-1* in a dose-dependent manner. Meanwhile, the molecular docking results showed that DSF and its derivatives could directly bind to MCR-1 (Fig. 4b, Supplementary Fig. 7, Supplementary Table 8), a rare member of lipid A phosphoethanolamine transferases (EptA)^25^. Therefore, we speculated that down-regulated *mcr-1* expression and the interaction of DSF-MCR contribute to impairing the action of MCR protein, thereby enhancing the electrostatic interaction of colistin and bacterial membrane. Next, scanning electron microscopy (SEM) analysis was performed to evaluate bacterial membrane damage under the DSF-colistin combination. We found that *E. coli* B2 cells in the control group, as well as colistin and DSF acting alone, showed regular and intact surfaces. In contrast, cells treated with a combination of colistin and DSF displayed severe cellular damage, including irregular and collapsed surfaces (Fig. 4c), suggesting that the mechanisms of this combination were associated with bacterial membrane rupture.

Next, the membrane permeability under exposure to colistin ranging from 0 to16 μg/mL alone or in combination with DSF (64 μg/mL) was determined using a fluorescent probe 1-*N*-phenylnaphthylamine (NPN). Consistent with the SEM results, the membrane permeability of *E. coli* B2 was significantly increased in the combination group compared with the colistin only (Fig. 4d). To further characterize membrane damage caused by the combination treatment, we evaluated the membrane potential and intracellular ROS levels using 3,3’-dipropylthiadicarbocyanine iodide (DiSC_3_(5)) and 2’,7’-dichlorodihydrofluorescei diacetate (DCFH-DA), respectively. As presented in Fig. 4e, f, compared with the colistin group, the supplementation of DSF dissipated membrane potential and boosted the oxidative damage of colistin on *E. coli* B2. These results indicate that DSF blocks the function of MCR protein and restores colistin damage to bacterial membranes, thereby resensitizing drug-resistant bacteria to colistin.

**Fig. 5:**
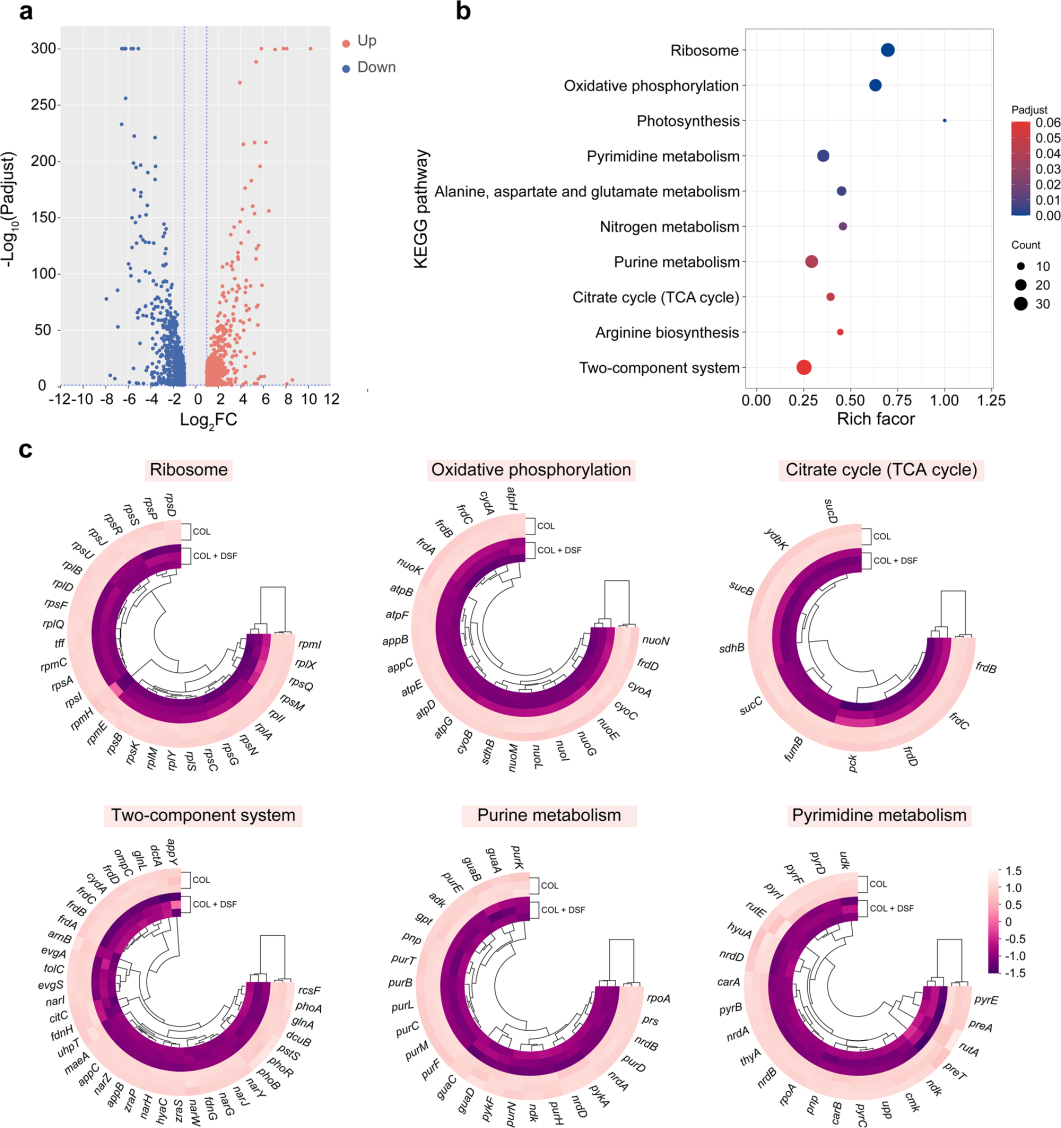
Transcriptomic analysis of E. coli B2 after treatment with colistin plus DSF in comparison with colistin alone. **a** Volcano plot of the differential expression genes (DEGs) in *E. coli* B2 after exposing to colistin alone (2 μg/mL) or in combination with DSF (64 μg/mL) for 4 h. The x and y axis in A represent the expression changes and corresponding statistically significant degree, respectively. An adjusted *P* value < 0.05 (Student’s *t*-test with Benjamini-Hochberg false discovery rate adjustment) and |log_2_ Fold change | ≥1 was applied as the cut-off for significant DEGs. **b** KEGG (Kyoto Encyclopedia of Genes and Genomes) enrichment analysis of down-regulated DEGs. The 10 most significantly enriched pathways were shown. **c** Cluster analysis of selected DEGs involved in ribosome, oxidative phosphorylation, TCA cycle, two-component system, purine metabolism and pyrimidine metabolism. Data were presented as means of three biological replicates. COL, colistin alone; COL + DSF, the combination of colistin and DSF.

To further investigate and elucidate the effect of the DSF-colistin combination on *mcr*-positive bacteria, we conducted RNA-sequencing analysis to compare the differentially expressed genes (DEGs) of *E. coli* B2 under DSF-colistin combination (64 + 2 μg/mL) treatment versus colistin alone (2 μg/mL). The volcano plot (Fig. 5a) showed that a total of 1368 DEGs (667 upregulated and 701 downregulated) with highly significant expression patterns under colistin treatment without/with DSF were identified. Surprisingly, the enrichment analysis of KEGG pathways showed no appreciable upregulation of enriched pathways. Besides, DEGs associated with ribosome, oxidative phosphorylation, pyrimidine metabolism, purine metabolism, citrate cycle (TCA cycle), and two-component system were significantly downregulated by the DSF-colistin combination compared with colistin alone (Fig. 5b). RT-qPCR assay was further conducted to validate transcriptional changes induced by the combination. Genes associated with ribosome, oxidative phosphorylation and pyrimidine metabolism were carefully examined, and a relatively high consistency was observed (Supplementary Fig. 4). The selected candidate genes display differential expression of at least two-fold compared to that in the colistin-alone group.

In particular, TCA cycle plays a vital role in the cellular respiration of bacteria and provides numerous critical intermediates required for other vital metabolic processes, including succinate and citrate^26^. However, the gene expression levels of *frdA*, *frdB*, *frdC*, *frdD* encoding fumarate reductase (FRD), which could reduce fumaric acid to succinic, were significantly downregulated. Citrate lyase required for citrate fermentation by *E. coli* is controlled by the *citCDEFXGT* gene cluster in the CitA/CitB two-component system. The genes controlling the synthesis of citrate did not alter substantially, while *citC* in the gene cluster was markedly downregulated.

Oxidative phosphorylation is also one of the pathways affecting bacterial central metabolism. Compared with those treated with colistin alone, genes encoding Nuo (*nuoE*, *nuoG*, *nuoI*, *nuoK*, *nuoL*, *nuoM*), the most efficient of the four NADH dehydrogenases, were significantly repressed by the DSF-colistin combination, suggesting a lowered redox state. In addition, the expression of the ATP synthase genes (*atpB*, *atpD*, *atpE*, *atpG*, *atpH*) was also decreased. Collectively, the inhibition of central carbon metabolites reduces electron transfer and ATP synthesis, which makes bacteria more susceptible to destruction. We also observed the inhibition of ribosome and nucleotide metabolism, corresponding to translation and transcription, respectively (Fig. 5c). Overall, we conclude that the combination treatment leaves the bacteria in a state of imminent death, where core processes and energy metabolism are both inhibited, in line with the previous results demonstrating their potent bactericidal effect.

### DSF thwarts the evolution and transmission of meropenem and colistin resistance

**Fig. 6:**
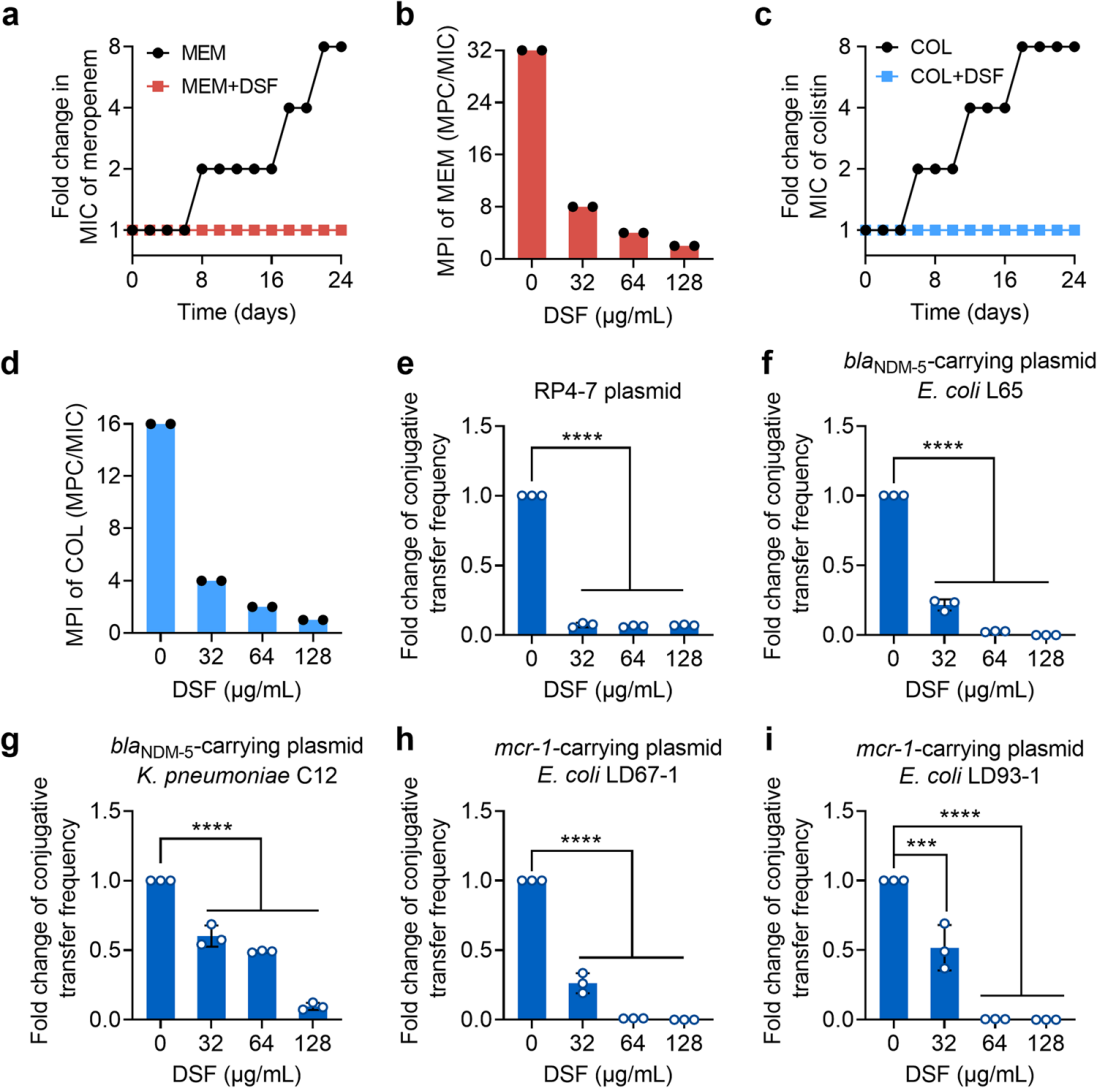
DSF thwarts the evolution and transmission of meropenem and colistin resistance. **a**, **c** Resistance development curves during serial passage of NDM-positive *E. coli* C3 (**a**) or MCR-positive *E. coli* G92 (**c**) in the presence of sub-MICs of meropenem/colistin or in the combination of DSF, respectively. **b**, **d** MPI indices of meropenem/colistin in the presence of increasing concentrations of DSF against NDM-positive *E. coli* C3 (**b**) or MCR-positive *E. coli* G92 (**d**), respectively. **e** Fold change of conjugative transfer frequency of RP4-7 plasmid from *E. coli* DH5α to *E. coli* EC600 in the presence of increasing concentrations of DSF. **f**–**i** Fold changes of conjugative transfer frequency *bla*_NDM-5_-positive plasmids from *E. coli* L65 (**f**) and *K. pneumoniae* C12 (**g**), *mcr-1*-positive plasmids from *E. coli* LD67-1 (**h**) and *E. coli* LD93-1 (**i**) to *E. coli* EC600 under different concentrations of DSF. Data in (**e**–**i**) were expressed as mean ± SD of three biological replicates. Statistical significance was analyzed by one-way ANOVA (***P < 0.001, ****P < 0.0001).

To learn more about whether the addition of DSF contributes to preventing the development of antibiotic resistance, we carried out the serial passage assays in an NDM- or MCR-positive bacteria after exposure to meropenem/colistin, DSF and their combination. Our results showed that meropenem or colistin resistance remained unaltered when combined with DSF, whereas meropenem or colistin alone both increased MICs by 8-fold throughout 24 serial passages, indicating that DSF prevents the evolution of drug resistance (Fig. 6a, c, Supplementary Table 9). The mutant prevention concentration (MPC) is an antibacterial threshold concentration where bacteria only can grow with two, or more than two, mutations, limiting the selective proliferation of mutant strains. The mutation prevention index (MPI) was determined via the equation of MPC/MIC, which represents the opening of the mutant selection window (MSW, concentration range between MIC and MPC). When the MSW is narrower, drug-resistant strains are less likely to develop^27^. As shown in Fig. 6a–d, in the presence of DSF, the MPI of meropenem and colistin were significantly decreased from 32 and 16 to 2 and 1, respectively, suggesting that the addition of DSF makes bacteria less likely to be resistant to drugs. The combined data clearly demonstrate that DSF restricts the development of meropenem and colistin resistance and the enrichment of mutant subpopulations.

As one of the major pathways of horizontal gene transfer (HGT), conjugation plays an essential role in the acquisition and transmission of antibiotic resistance genes (ARGs)^28^. Therefore, we conducted a series of conjugation assays to determine the impact of DSF on resistance plasmids transfer. Compared with the control, the conjugation frequency of RP4-7 plasmid was significantly reduced under the treatment of DSF (Fig. 6e). To further investigate the effect of DSF in clinical isolates, we performed conjugation experiments with clinical isolates harboring various types of plasmids carrying *mcr-1* and *bla*_NDM-5_. Consistently, DSF successfully inhibited the transmission of *bla*_NDM-5_-positive IncX3 plasmids from donors to recipients (Fig. 6f, g). Similarly, the transmission of *mcr-1*-carrying IncX4 pLD93-1 and IncI2 pLD67-1 plasmids from *E. coli* LD67-1 and LD93-1 to *E. coli* EC600 was obviously decreased in the presence of DSF (Fig. 6h, i). These results indicated the potential of DSF in preventing the spread of ARGs-carrying plasmids. Collectively, these data demonstrate that DSF could minimize the evolution and spread of meropenem and colistin resistance.

### DSF rescues meropenem and colistin efficacy in vivo

**Fig. 7:**
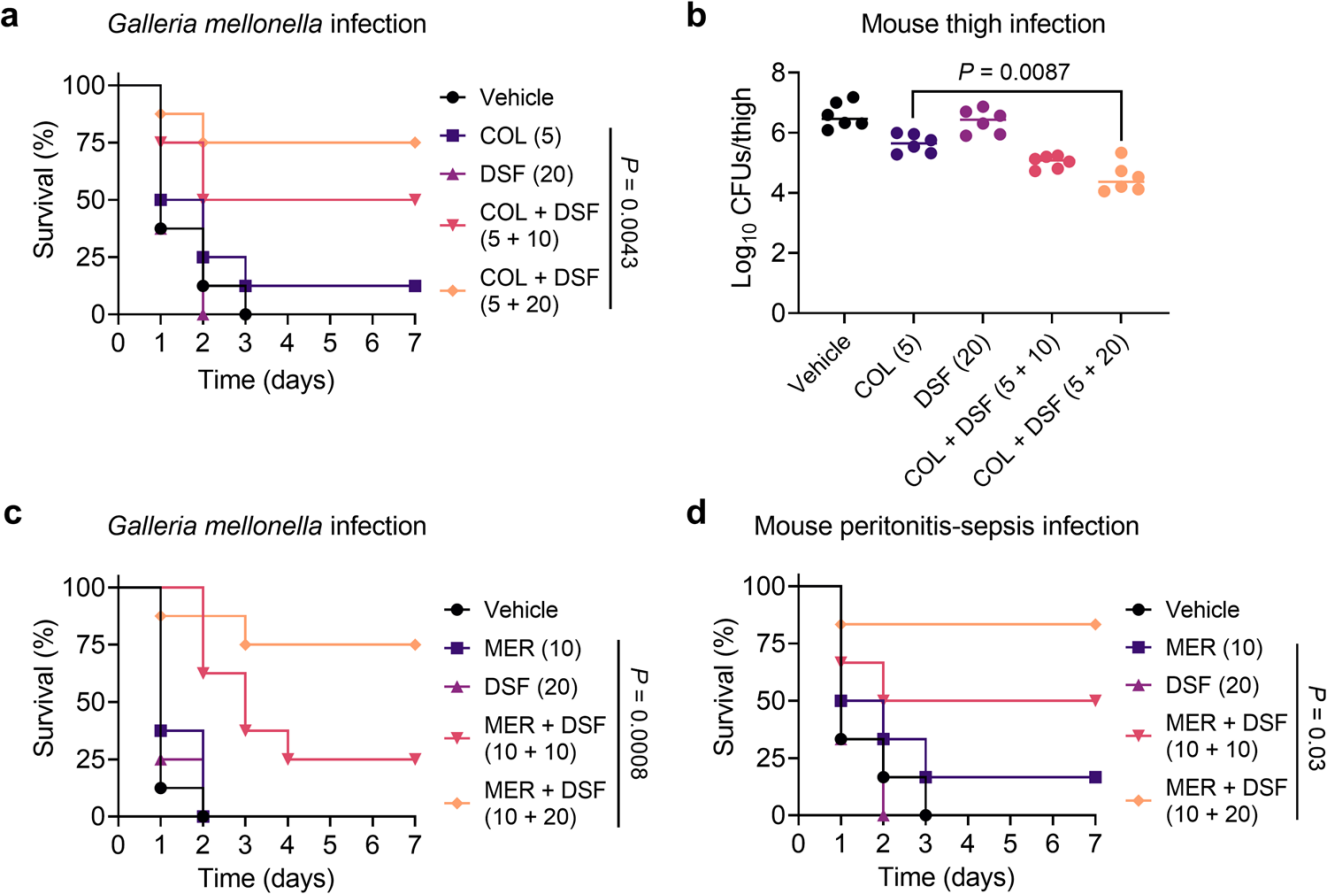
DSF improves the in vivo efficacy of colistin and meropenem. **a** Survival rates of the *G. mellonella* larvae (*n* = 8 biologically independent animals per group) infected by *E. coli* B2 (10^6^ CFUs). Compared with colistin monotherapy (5 mg/kg), the combination therapy of colistin and DSF (5 + 20 mg/kg) significantly improved the survival rates of *G. mellonella*. **b** Bacterial loads of the CD-1 mice (*n* = 6 biologically independent animals per group) infected by *E. coli* B2 (10^5^ CFUs). Compared with colistin alone, the combination therapy of colistin and DSF reduced bacterial loads of thigh muscle. *P*-values were determined by Mann-Whitney *U* test. **c** Survival rates of the *G. mellonella* larvae (*n* = 8 biologically independent animals per group) infected by *E. coli* C3 (10^6^ CFUs). Compared with the meropenem monotherapy (10 mg/kg), the combination therapy of meropenem and DSF (10 + 20 mg/kg) significantly improved the survival rates of larvae. **d** Survival rates of the CD-1 mice (*n* = 6 biologically independent animals per group) infected by *E. coli* C3 (10^8^ CFUs) and treated with a single dose of meropenem (10 mg/kg), DSF (20 mg/kg), a combination of meropenem plus DSF (10 + 10 mg/kg, 10 + 20 mg/kg), or PBS by intraperitoneal injection. In (**a**), (**c**) and (**d**), *P* values were determined by log-rank (Mantel-Cox) test.

Prior to the in vivo effectiveness evaluation, we performed a series of safety experiments to assess the toxicity of the combination of DSF and colistin, including hemolytic activity on mammalian RBCs and in vivo toxicity in mice. Interestingly, the combined use of DSF (0 to 128 μg/mL) and colistin (2 to 16 μg/mL) displayed a low hemolysis rate (<10%) (Supplementary Fig. 5). Furthermore, compared with the vehicle group, no toxicity was observed in mice models co-administrated with colistin and DSF (Supplementary Fig. 8). Next, a panel of preclinical infection models was conducted to explore the therapeutic potential of combination treatment in eradicating MDR pathogens. The results indicated that co-administration of colistin with DSF (5 + 20 mg/kg) greatly prevented the death of the group of the *G. mellonella* infected by *mcr*-positive *E. coli* compared with colistin monotreatment group (*P* = 0.0043) (Fig. 7a). Thereafter, we evaluated the combinational efficacy in a neutropenic mouse thigh infection model. As shown in Fig. 7b, the combinational therapy of colistin and DSF (5 + 20 mg/kg) exhibited nearly 2-log_10_ CFUs reduction compared with colistin monotherapy (*P* = 0.0087). Furthermore, DSF was also proven to restore the in vivo efficacy of meropenem in two animal models of infection. Conceivably, the larvae in the vehicle group and meropenem alone all died within 48 h. On the contrary, the survival rates of *G. mellonella* in the meropenem plus DSF group (10 + 20 mg/kg) was obviously increased and achieved a 75% survival rate during 7 d post-infection (*P* = 0.0008) (Fig. 7c). Lastly, we tested the combinational efficacy in a mouse peritonitis infection model, and we found that meropenem-DSF treatment successfully obtained survival benefits compared with the meropenem group (*P* = 0.03) (Fig. 7d). Collectively, these data demonstrate that DSF effectively restores the in vivo efficacy of meropenem and colistin against drug-resistant bacterial infections.

## Discussion

The increasing incidence of drug-resistance infections, particularly caused by Gram-negative pathogens, seriously threatens public health. Carbapenems and colistin are the last resorts for the treatment of hard-to-treat bacterial infections in clinic. However, the emergence of plasmid-mediated bacterial resistance to these last options leaves no available regimens in clinical settings. Compared with the discovery of new antibiotics, antibiotic adjuvants strategy is recognized as a more cost-effective approach to tackling MDR pathogens. For example, our previous studies found that melatonin could serve as a promising colistin adjuvant^4^, a hypoglycemic agent metformin^29^ and non-steroidal anti-inflammatory drug (NSAID) benzydamine^30^ are potential tetracyclines adjuvants, anti-HIV drug azidothymidine^31^ restores tigecycline activity against *tet*(X)-positive *E. coli*. However, these antibiotic adjuvants were found to act primarily on one or a class of antimicrobial agents. Here, we revealed that an FDA-approved drug DSF and its derivates can serve as versatile and potent antibiotic adjuvants to overcome MDR Gram-negative bacteria. In particular, DSF drastically potentiated meropenem activity against a range of carbapenem-resistant Enterobacteriaceae (CRE), and colistin activity against MCR-positive pathogens both in vitro and in animal models of infection. Notably, the potentiation of DSF to colistin was robust, accompanied by lower FICI compared with other colistin synergists^9^. Most importantly, our study also found that the addition of DSF prevented the evolution of bacterial resistance to meropenem and colistin and inhibited the conjugative transfer of *bla*_NDM_ and *mcr*-carrying plasmids.

With regard to the potentiation of DSF to carbapenems, our mechanistic studies demonstrated that DSF could specifically inhibit NDM activity, thereby potentiating meropenem efficacy. This mode of action explains why the synergistic activity of meropenem and DSF were only found in NDM-positive bacteria, but not NDM-negative bacteria. Consistent with our findings, a previous study also indicated that DSF could serve as a potent metallo-β-lactamase inhibitor by forming an S-S bond with the Cys208 residue of NDM and oxidizing its Zn(II) thiolate site^24^. As for the potent synergistic effect between DSF and colistin, our results revealed that DSF could simultaneously inhibit mRNA expression of *mcr-1* and bind to MCR enzymes, thereby restoring membrane damage ability of colistin. Consistently, the combined treatment of DSF and colistin exacerbated cell membrane damage, increased membrane permeability, and resulted in the dissipation of membrane potential. The inhibition of central metabolism and nucleotide metabolism by colistin-DSF treatment appears to be a result underlying the synergistic killing activity. Previous studies have shown that inhibition of antibiotic targets could result in downstream metabolic perturbations^32^, and colistin can differentially alter the levels of metabolites associated with central carbon metabolism, a new target for antimicrobial drugs^10,26,33–35^. In the present study, we also showed that colistin-DSF combination drastically perturbed the central carbon metabolic pathways, including TCA cycle and oxidative phosphorylation.

Nowadays, DSF is used clinically for the treatment of alcohol dependence, and some studies have indicated that it is also a potent anticancer agent^36^ and pyroptosis inhibitor^37^. It is suggested that DSF has an acceptable risk profile and there is no available information on the overdose treatment of DSF so far. Also, our results demonstrated that the DSF-colistin combinations displayed negligible hemolysis to mammalian RBCs and no acute toxicity in mice models. However, substantial clinical trials are still required to evaluate the clinical efficacy of this combination and whether it has dangerous side effects in humans.

In conclusion, we demonstrate that DSF and its derivates remarkably restore the susceptibility of NDM- and MCR-positive superbugs to meropenem and colistin, respectively. DSF acts mainly by inhibiting the enzymatic activity of resistance proteins. Moreover, DSF slows down the evolution and transmission of drug resistance. Importantly, DSF combined with meropenem or colistin effectively combat MDR bacterial infections in vivo, highlighting its therapeutic potential as a promising antibiotic potentiator.

## Methods and materials

### Bacterial strains and growth conditions

Strains used in this study were presented in Supplementary Table 1. Bacteria were stored in nutrient broth supplemented with 20% glycerol at −80 °C. Unless otherwise noted, LB broth or LB agar was used for the normal growth of all bacteria.

### Minimal inhibitory concentration (MIC) assay

MICs determination of drugs was based on the standard microdilution method according to the CLSI 2021 guideline^38^. Briefly, compounds were two-fold serial dilution with MHB. Next, log-phase bacteria suspensions were adjusted to 1 × 10^6^ CFUs/mL and mixed with compounds in a 96-well microliter plate (Corning, NY, USA). Plates were incubated at 37 °C for 24 h, and the MIC values were detected as the lowest concentration of drugs with no visible bacterial growth.

### Checkerboard assay

Synergistic activities of drugs were tested by checkerboard assays^39^. Briefly, 100 µL of MHB was dispensed into a 96-well microliter plate. Colistin and compounds were created an 8 × 8 matrix with eight two-fold serially dilution. Bacterial culture was grown to log phase and diluted to 1 × 10^6^ CFUs per mL. After 18 h incubation at 37 °C, the absorbance of each well at 600 nm was measured by a Microplate reader (Tecan, Männedorf, Switzerland). The fractional inhibitory concentrations index (FICI) was calculated accordingly. FICI ≤ 0.5 demonstrates synergy.

### Time-dependent killing

Overnight bacteria culture was diluted 1:1000 into LB and incubated for 4 h at 37 °C with 200 rpm. Then, the culture was treated by either PBS, colistin, DSF or colistin in combination with DSF. At the time points 0, 6, 12 and 24 h, 100 µL bacteria culture was removed and resuspended in PBS, and the serial dilutions were spotted on LB agar. After incubation overnight at 37 °C, the colony counts were collected.

### Biofilm formation and eradication assay

The effect of DSF on biofilm formation and eradication when in combination with colistin was evaluated according to our previous study^30^. In brief, crystal violet staining method was used to monitor the formation of biofilm. *E. coli* B2 mixed with colistin alone or in combination with DSF were cultured in 96-well microtiter flat plate (Corning) at 37 °C for 36 h. Then, the planktonic bacteria were removed by washing three times with sterile PBS solution and 100 µL methanol was added and fixed for 15 min. After aspirating the fixative and natural air drying, dried wells were stained with 100 µL of 0.1% crystal violet for 15 min and the remaining crystal violet was rinsed with PBS for twice. Finally, 100 µL 33% acetic acid was added and cultured at 37 °C for 30 min to dissolve crystal violet. Biofilm mass was determined at an absorbance of 570 nm using a microplate reader (Tecan Infinite E Plex).

Biofilm eradication effect was assessed by counting the bacterial colonies. Exponential phase bacteria cells were diluted in 1/1,000 with MHB and cultured in a 96-well microtiter plate (Corning) at 37 °C for 48 h to promote biofilm formation. After washing three times with PBS, colistin alone or colistin-DSF combination were added and cultured at 37 °C. After 2 h of incubation, the wells were emptied, washed, and sonicated for 15 min to disperse biofilm cells. Next, bacteria-diluted suspensions were plated on MHA plates and incubated overnight at 37 °C. Bacterial colonies were counted and the primary CFUs per mL were calculated. Finally, the remaining CFUs were used to evaluate the removal of the biofilm.

### Protein purification and enzyme inhibition assay

Protein purification and enzyme inhibition assays were performed according to previous studies^40,41^. Briefly, cloned expression vector with coding sequence of NDM-1 or NDM-5 was chemically transformed into *E. coli* BL21 (DE3). The recombinant NDM proteins were purified using NTA (nitrilotriacetic acid) agarose and then assessed by SDS-PAGE analysis, and its concentrations were measured using an enhanced BCA protein assay kit (Beyotime, Shanghai, China). Purified NDM proteins (50 nM) in HEPES buffer (50 mM, pH = 7.2) were incubated with increasing concentrations of DSF for 5 min at 25 °C. Enzyme inhibition assay was performed in a 96-well plate at an absorbance of 492 nm at room temperature by a microplate reader (Tecan Infinite E Plex). Positive controls were performed in the presence of NDM and in the absence of DSF, whereas negative controls were performed in the absence of NDM. The residual activity of NDM was calculated as follows:

(Equation 1) Residual activity (%) = (OD_sample_ − OD_negative_) / (OD_positive_ – OD_negative_) × 100%

### Molecular docking

The chemical structure of DSF was prepared using ChemDraw software. The crystal structures of NDM-1 (PDB code, 4EYL) or MCR-1 (PDB code, 5GRR) protein was obtained from the Protein Data Bank (PDB), and DSF was preprocessed by Pymol software. Docking was subsequently performed using the Ledock software. The best-generated models were chosen for interaction analysis. All figures for the structure models were generated using Pymol software.

### RT-qPCR analysis

*E. coli* B2 was grown overnight and diluted 1:1,000 into the fresh medium with different concentrations of DSF. After bacterial cells were grown to an OD_600_ of 0.5, total RNA was extracted using the TRIzol reagent (Invitrogen) according to the manufacturer’s instructions. Then, a total of 1000 ng of purified RNA was reverse transcribed into cDNA using PrimeScript RT reagent Kit with gDNA Eraser (Takara, Beijing, China). The following PCR reaction mixture was prepared using the ChamQ™ SYBR® Color qPCR Master Mix Kits (Vazyme Biotech, Nanjing, China) and the RT-qPCR analysis was conducted by 7500 Fast Real-Time PCR System (Applied Biosystem, CA, USA). The amplification parameters were set as follows: initial denaturation at 95 °C for 30 s, followed by 40 cycles at 95 °C for 5 s, 60 °C for 30 s, and 72 °C for 45 s. The expression of 16 s rRNA served as the housekeeping gene to analyze the changes in other genes using 2^-(∆∆Ct) method. The primer sequences were listed in Supplementary Table 6.

### Scanning electron microscopy

The SEM was used to evaluate the effect of DSF on bacterial surface morphology^42^. Briefly, bacteria cells were grown to exponential phase and pre-incubated with colistin alone or a combination of DSF and colistin at 37 °C. After incubation for 1 h, the cells were washed twice with PBS, and then fixed with 2.5% glutaraldehyde at 4 °C overnight. Subsequently, the samples were dehydrated in an ethanol series (30%, 50%, 70%, 90 and 100%) for 10 min. The samples were evaluated with GeminiSEM 300.

### Membrane permeability assay

The outer membrane permeability of *E. coli* B2 treatment by DSF was measured with fluorescent probe 1-*N*-phenylnaphthylamine (NPN, 10 μM)^43^. Bacterial cells were washed with PBS and adjusted to 1 × 10^7^ CFUs/mL, and then the bacterial suspension was incubated with NPN at 37 °C for 30 min. Next, the mixture was added to black 96-well plates (Corning, NY, USA) containing the serial concentration of drugs. Fluorescence intensity was determined with excitation and emission wavelengths at 350 and 420 nm.

### Cell membrane integrity assay

The cell membrane integrity of *E. coli* B2 was measured with 10 nM propidium iodide (PI)^44^. The fluorescence was evaluated with the excitation wavelength at 535 nm and emission wavelength at 615 nm.

### Membrane depolarization assay

The fluorescent dye 3,3’-dipropylthiadicarbocyanine iodide (DiSC_3_(5)) was used to evaluate the cytoplasmic membrane depolarization of *E. coli* B2 treated by DSF^45^. Bacterial cells were resuspended to an OD_600_ of 0.5, and then DiSC_3_(5) was added to a final concentration of 0.5 μM and incubated for 10 min. After 30 min, bacterial suspension was injected to a black-walled plate, and a final concentration of DSF or increasing concentrations of colistin was added. The fluorescence intensity was determined with excitation/emission wavelengths of 622/670 nm.

### ROS measurement

The ROS level was tested as described previously. Log-phase *E. coli* B2 was incubated with 2’,7’-dichlorodihydrofluorescein diacetate (DCFH-DA) (10 μM) for 30 min, the bacteria cells were washed three times with PBS to ensure the removal of excess fluorescent probes. Next, the probed-cells were treated with varying concentrations of colistin and DSF for 1 h. After incubation, the fluorescence intensity was immediately measured with excitation and emission wavelengths at 488 and 525 nm.

### Transcriptomic analysis

Colistin-resistant *E. coli* B2 was grown to the exponential phase, and bacteria were cultured with colistin alone or in combination with DSF (*n* = 3 biologically independent samples). After incubation for 4 h, cells were collected, and total RNA of bacteria was extracted using the TRIzol reagent (Invitrogen). Next, the RNA was quantified by using a Nanodrop spectrophotometer (Thermo Scientific, MA, USA), followed by library construction, sequencing and bioinformatics analysis. The RNA-seq library was constructed using the Illumina® Stranded mRNA Prep kit (San Diego, CA). Briefly, all mRNAs were broken into short (200 nt) fragments by adding fragmentation buffer. Then, double-stranded cDNA was synthesized with random hexamer primers (Illumina) and subjected to end-repair, phosphorylation and adenine addition according to Illumina’s library construction protocol. Paired-end RNA-seq library was sequenced with the Illumina Novaseq 6000 (Illumina Inc., San Diego, CA, USA). Subsequently, the data generated from Illumina platform were used for the following bioinformatics analysis. All of the analyses were performed using the free online platform of Majorbio Cloud Platform (www.majorbio.com). The DESeq2 package was used for the differential expression analysis. Functional annotation was performed with GO and KEGG database.

### Resistance development assay

The resistance development of a combination of colistin and DSF was evaluated using a previously described method^46^. Briefly, log-phase NDM-positive *E. coli* C3 or MCR-positive *E. coli* G92 was diluted in LB broth supplement with 0.25 × MIC of DSF in the presence or absence of colistin (0.25 × MIC) and cultured at 37 °C for 24 h. the MIC of bacteria was determined. Next, this culture was diluted into fresh LB broth containing sub-MIC of drugs for the next passage. The in vitro passage was repeated continuously for 24 days.

### Conjugation transfer frequency determination

Bacteria were grown in LB broth at 37 °C overnight to achieve an OD_600_ of 0.5. To form the final conjugation system, 1 mL donor and recipient bacteria were mixed and cultured for 15 h in the presence of different concentrations of DSF (0 to 128 μg/mL). Besides, several clinical strains with *bla*_NDM-5_-bearing plasmids and *mcr-1*-bearing plasmids were further determined. IncX3 *bla*_NDM-5_-bearing plasmids were from *K. pneumoniae* and *E. coli*, respectively. The two IncI2 and IncX4 plasmids with *mcr-1* resistance genes were derived from clinical isolates *E. coli* LD67-1 and LD93-1.

### Galleria mellonella infection

*Galleria mellonella* larvae infection model was established to evaluate the synergy between colistin/meropenem and DSF in vivo. Briefly, larvae (Huiyude Biotech, Tianjin, China) were randomly divided into six experimental groups (*n* = 8 biologically independent animals per group) and 10 µL of *E. coli* B2 or C3 suspension (10^6^ CFUs) was infected to the right posterior gastropoda. After incubation for 1 h, a single dose of colistin (5 mg/kg), meropenem (10 mg/kg), DSF (20 mg/kg), colistin plus DSF (5 + 10 mg/kg, 5 + 20 mg/kg), meropenem plus DSF (10 + 10 mg/kg, 10 + 20 mg/kg), or PBS was administered. Thereafter, survival rates were recorded until 7 days post-infection.

### Hemolysis analysis

For hemolysis analysis, the samples were processed as described previously^47^. Briefly, colistin plus DSF was equal-volume incubated with 8% fresh sterile defibrinated sheep blood cells at 37 °C. PBS was used as a negative control and ddH_2_O was used as a positive control. Hemolytic activity was measured at an absorbance of 567 nm by an Infinite E Plex Microplate reader (Tecan) and the hemolysis rate was calculated accordingly.

### Animal studies and ethical statement

Female CD-1 mice (aged 8 weeks; 20–25 g) were obtained from Comparative Medicine Center of Yangzhou University. Animal experiments were performed according to the guidelines of Jiangsu Laboratory Animal Welfare and Ethical of Jiangsu Administrative Committee of Laboratory Animals. The animal use protocols were approved by Jiangsu Laboratory Animals Administrative Committee (SYXK-2022-0044). The laboratory animal usage license number is SCXK-2022-0009, certified by the Jiangsu Association for Science and Technology.

### Safety assessment

The in vivo toxicity of DSF-COL combination was determined by intraperitoneally injecting a single dose of drug combination into CD-1 mice (*n* = 6 biologically independent animals per group). In addition to injections for 6 days, animal behaviors and body weight should also be continuously recorded. Blood was collected on the last day for blood biochemical assay and whole-blood cell analysis.

### Neutropenic mouse thigh infection

Briefly, 8-week-old CD-1 female mice were rendered neutropenic by delivering cyclophosphamide (150 and 100 mg/kg) on 4 and 1 d before injection (*n* = 6 biologically independent animals per group). Subsequently, 100 μL of exponential-phase *E. coli* B2 suspension (10^5^ CFUs per mouse) was injected into the right thighs. At 2 h post-infection, a single dose of colistin (5 mg/kg, *i.p*.), DSF (2 mg/kg, *i.p*.), colistin plus DSF (5 + 10 mg/kg, 5 + 20 mg/kg), or PBS was administered. Next, mice were euthanized after 48 h post-infection. The right thighs were aseptically collected, homogenized, serially diluted, and plated on LB agar. After incubation at 37 °C for 24 h, the number of bacteria in the tissues was counted.

### Mouse peritonitis infection

Female CD-1 mice (*n* = 6 biologically independent animals per group) were intraperitoneally infected with a dose of 10^8^ CFUs *E. coli* C3 suspension. At 2 h post-infection, mice were administrated with a single dose of meropenem (10 mg/kg), DSF (20 mg/kg) alone or a meropenem combination with DSF (10 + 10 mg/kg, 10 + 20 mg/kg) via intraperitoneal injection. Survival rates of treated mice were recorded during 7 days.

### Statistics and reproducibility

Statistical analysis was performed using GraphPad Prism version 9.0. All experiments were performed with at least three independent biological replicates and data are expressed as mean ± standard deviation (SD). One-way/two-way ANOVA was used to calculate *P*-values (*P < 0.05, **P < 0.01, ***P < 0.001 and ****P < 0.0001).

### Reporting summary

Further information on research design is available in the Nature Portfolio Reporting Summary linked to this article.

### Supplementary information

Supplementary information

Description of Additional Supplementary Files

Supplementary Data 1

Reporting Summary

## Data Availability

RNA-sequencing data have been deposited in the National Center for Biotechnology Information (NCBI) Sequence Read Archive (SRA) database (PRJNA817728). Source data for the main figures are provided in Supplementary Data 1. All other data are available from the corresponding authors.
