# Supplementary material for: Resensitizing multidrug-resistant Gram-negative bacteria to carbapenems and colistin using disulfiram
Source: Commun Biol. 2023 Aug 3;6:810. doi: 10.1038/s42003-023-05173-7 (PMC10400630; doi:10.1038/s42003-023-05173-7)
Supplement: Supplementary file 1 — Supplementary information [file 42003_2023_5173_MOESM1_ESM.pdf]

Supplementary Information

Content list

Supplementary Figures

Supplementary Figure 1 --1

Supplementary Figure 2 --2

Supplementary Figure 3 --3

Supplementary Figure 4 --4

Supplementary Figure 5 --5

Supplementary Figure 6 --6

Supplementary Figure 7 --7

Supplementary Figure 8 --8

Supplementary Tables

Supplementary Table 1 --9

Supplementary Table 2 --10

Supplementary Table 3 --11

Supplementary Table 4 --12

Supplementary Table 5 --13

Supplementary Table 6 --14

Supplementary Table 7 --15

Supplementary Table 8 --16

Supplementary Table 9 --17

Supplementary Figures

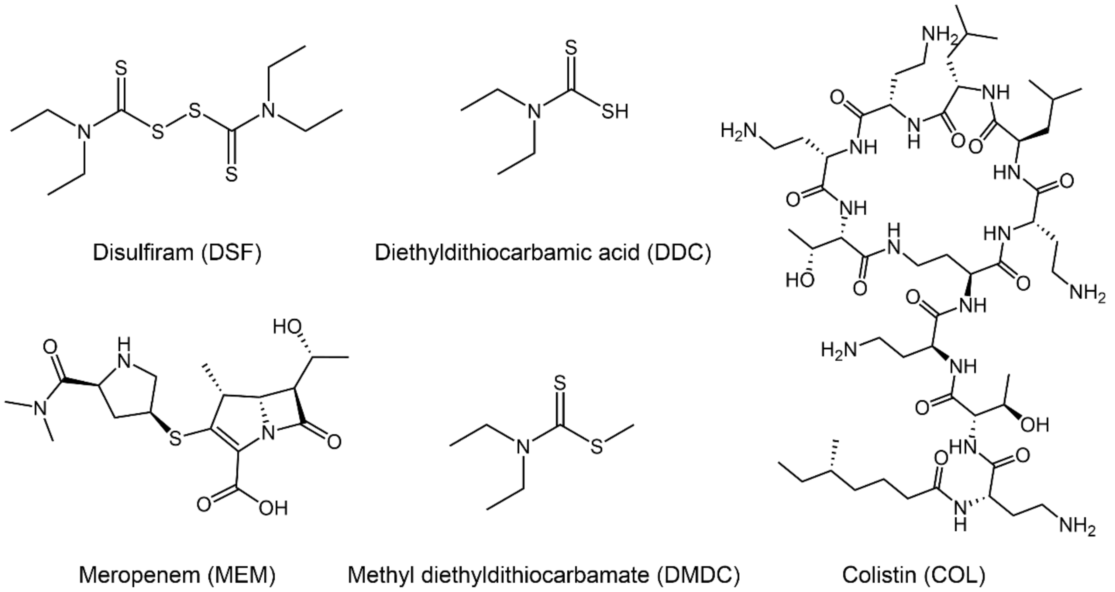

Supplementary Figure 1 Chemical structures of disulfiram (DSF), diethyldithiocarbamate (DDC), dimethyldithiocarbamate (DMDC), meropenem (MEM) and colistin (COL).

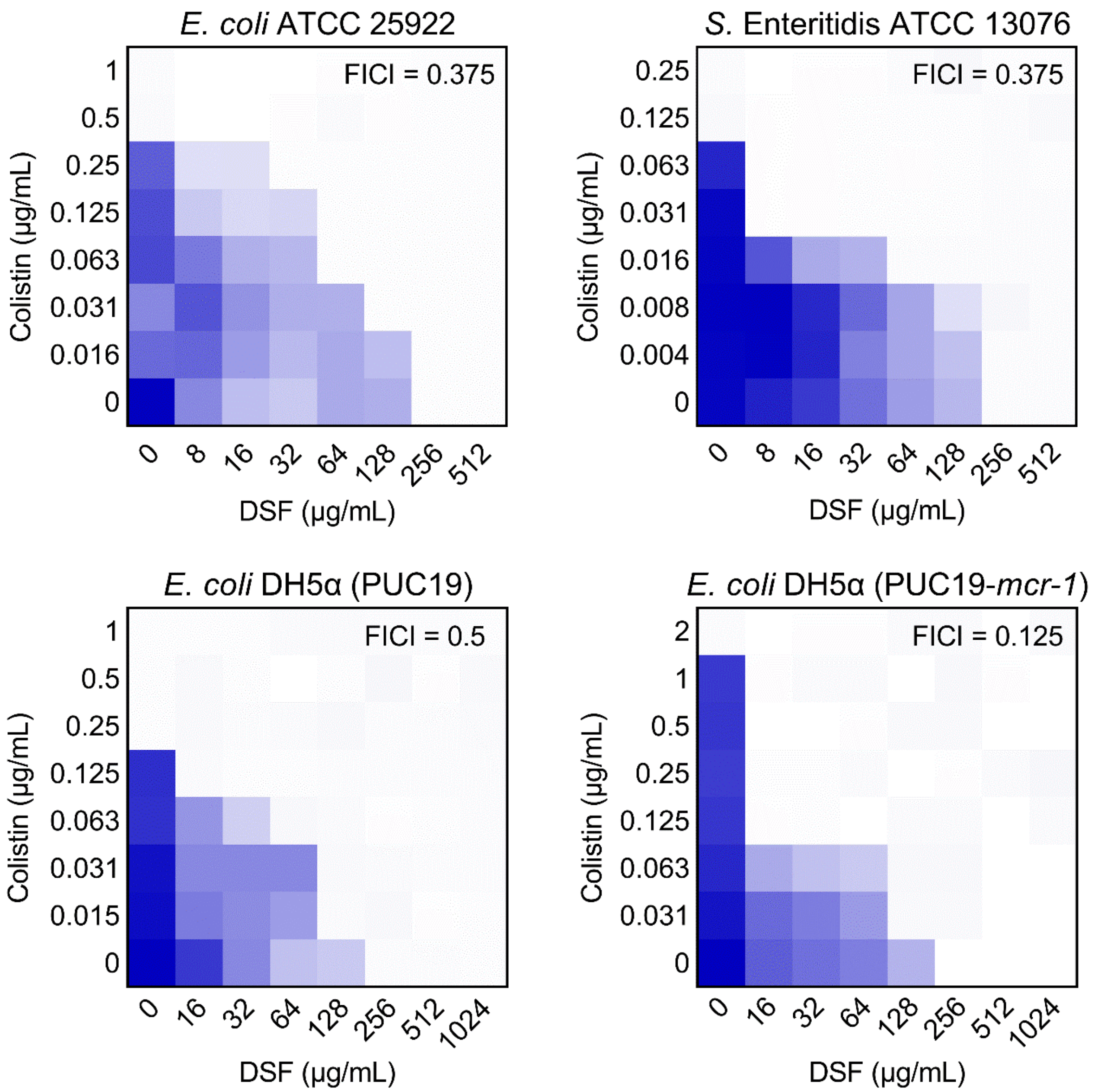

Supplementary Figure 2 Potentiation of DSF to colistin against *mcr-1*-negative and -positive bacteria, related to Supplementary Table 5.

Dark blue regions represent higher cell density. Data represent the mean OD (600 nm) of biological replicates.

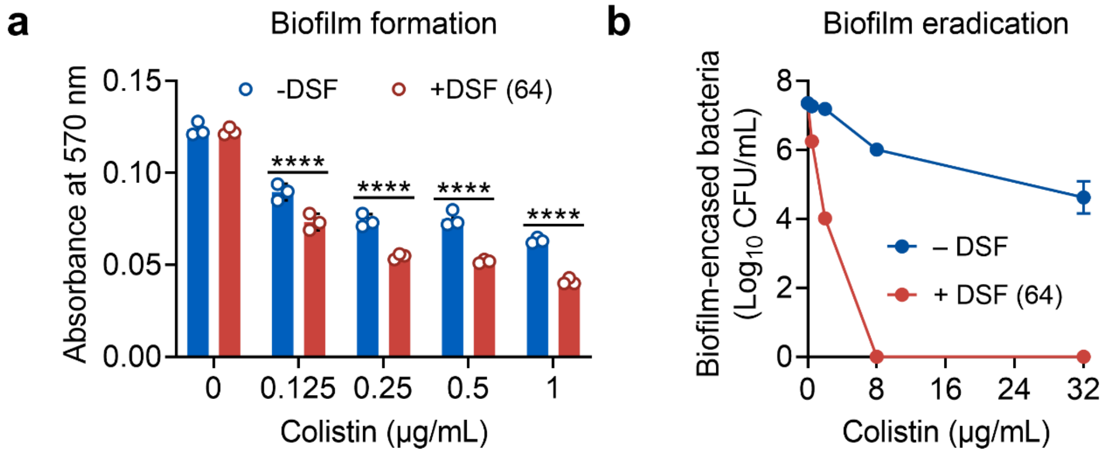

Supplementary Figure 3 Combination of colistin and DSF prevents the formation of biofilms (a) and eradicate the mature biofilm (b).

Data were presented as mean ± SD of three biological replicates. Statistical significance was analyzed by two-way ANOVA with Sidak's multiple comparisons test (*****P* < 0.0001).

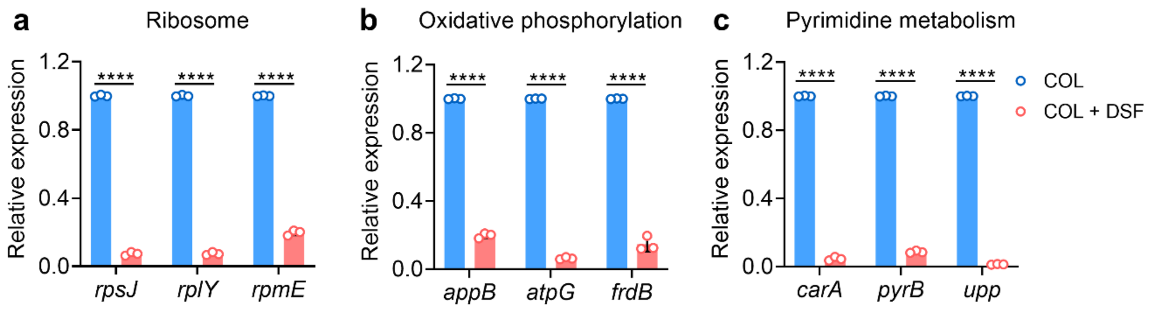

Supplementary Figure 4 Validation of transcriptomic results by RT-qPCR analysis. Data analysis was performed using 2^-(ΔΔCt) method, with 16S rRNA serving as the housekeeping gene. Experiments were conducted with three biological replicates. Data were presented as mean ± SD. Statistical significance was analyzed by two-way ANOVA with Sidak's multiple comparisons test (*****P* < 0.0001).

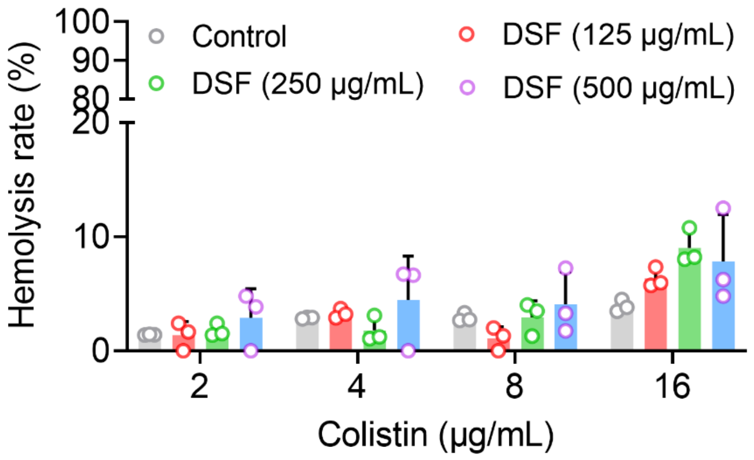

Supplementary Figure 5 Effect of DSF on hemolytic activity of colistin against mammalian red blood cells (RBCs).

PBS and ddH_2O were used as negative and positive controls, respectively. The ddH_2O was defined as 100% in the calculation of hemolysis rate. Data were presented as mean ± SD of three biological replicates.

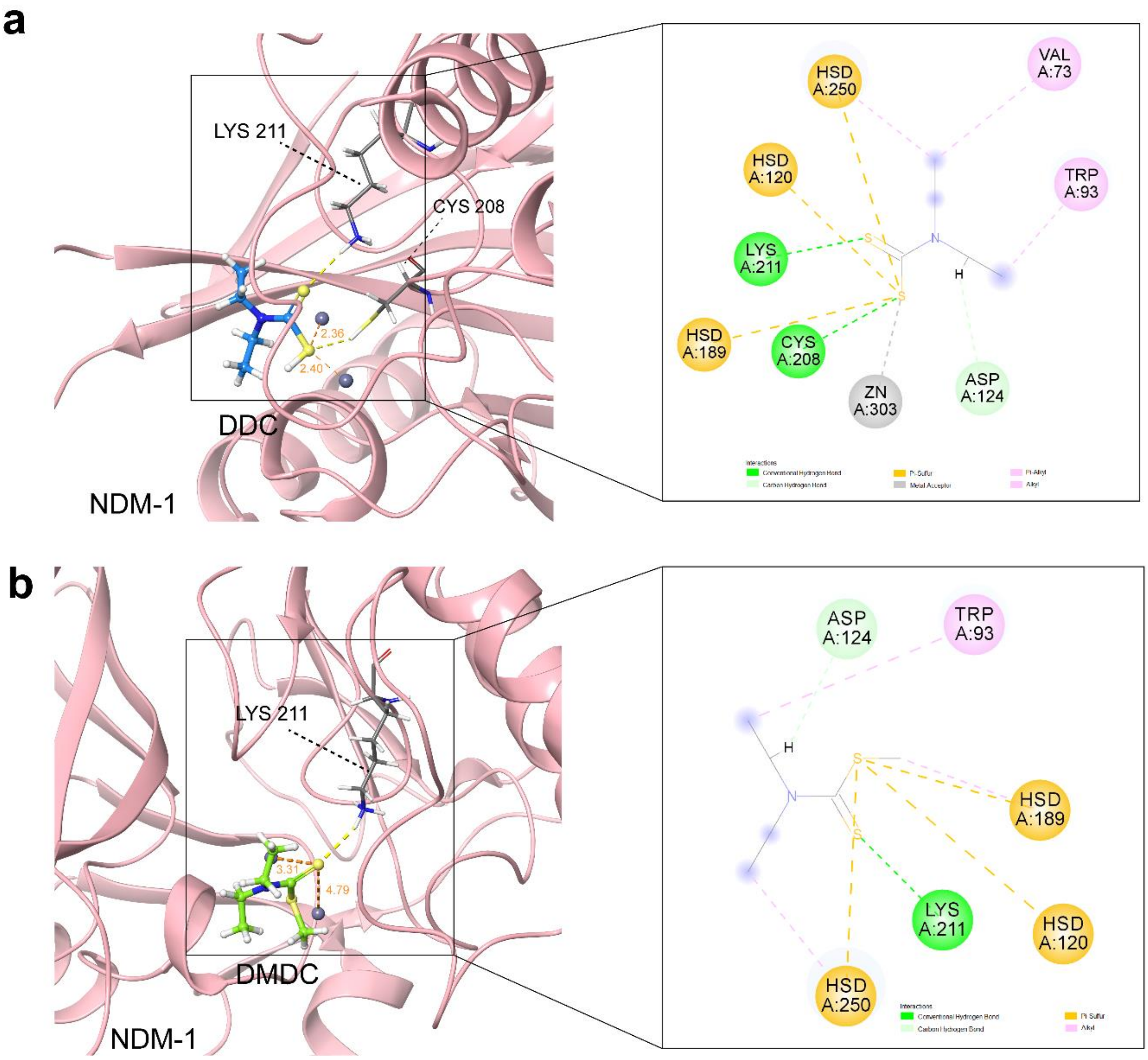

Supplementary Figure 6 Molecular docking analysis of the complexes of NDM-1 with DDC (a) and DMDC (b). The NDM-1 structure was represented as a pink ribbon.

(a) Overlay of DDC bound to NDM-1 protein. In the close-up 2D view, the hydrogen bonds formed between the ligand and the protein were depicted as dashed green lines, and the residues involved in the hydrogen bond formation include CYS 208 and LYS 211, with the coordination of one sulfur atom of DDC with Zn.

(b) Overlay of DMDC bound to NDM-1 protein. In the close-up 2D view, the residue involved in the hydrogen bond formation was LYS211 only.

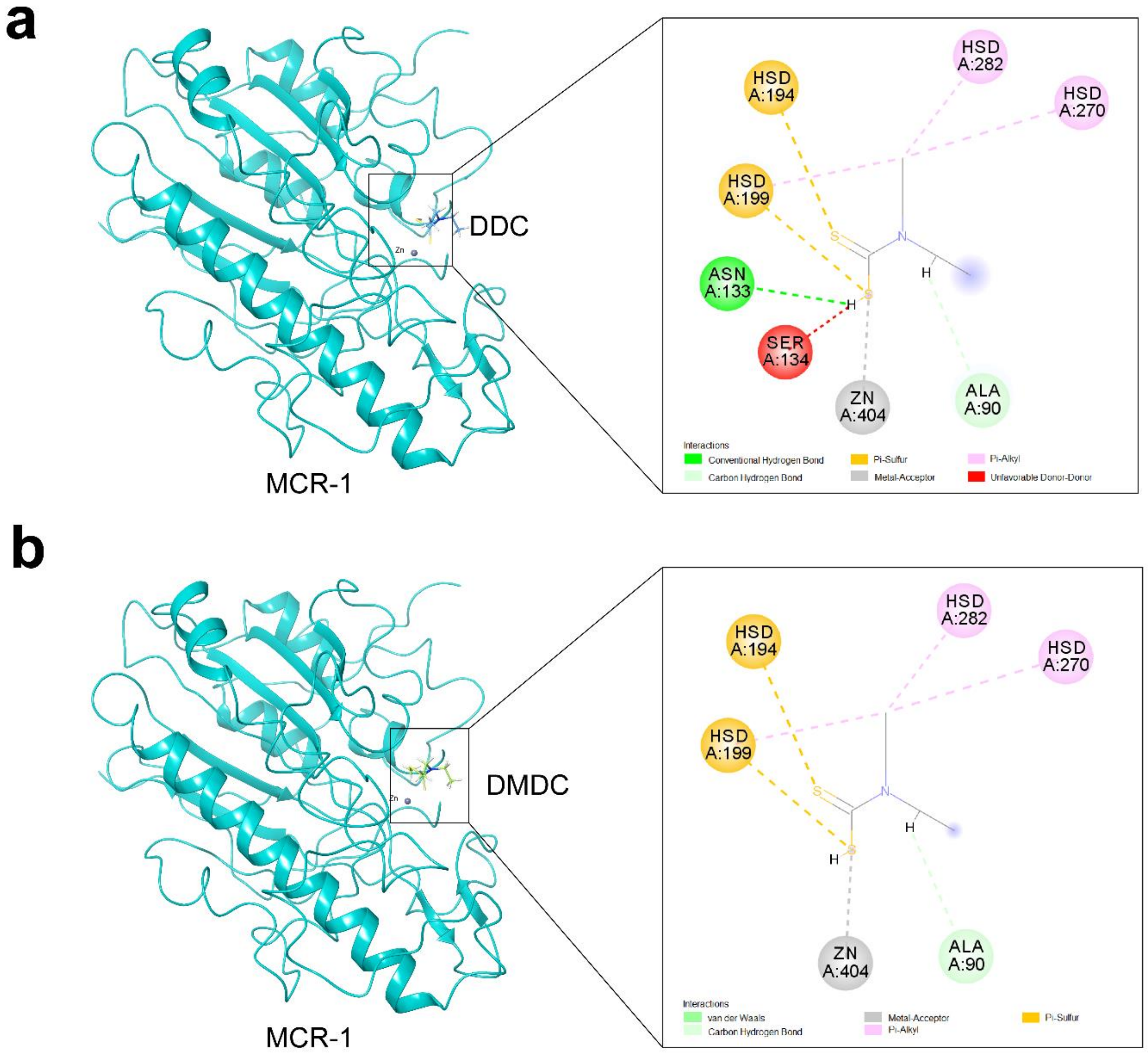

Supplementary Figure 7 Molecular docking analysis of the complexes of MCR-1 protein with DDC (a) and DMDC (b).

In the close-up view, the hydrogen bonds formed between them were depicted as dashed green lines, and the compound involved in the metal coordination formation with the active-site zinc ion.

a

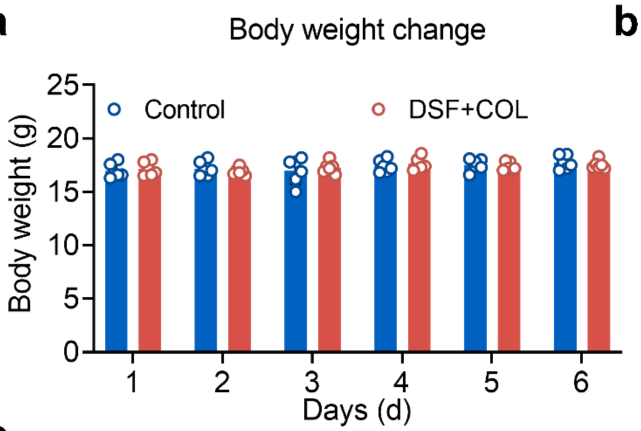

b

Parameters	Unit	Control	DSF+COL
WBC	10^9cells/L	6.3	6.7
LYM	10^9cells/L	4.6	5.0
MON	10^9cells/L	0.2	0.2
GRAN	10^9cells/L	1.5	1.5
LYM	%	79.1	78.2
MON	%	2.3	2.5
GRAN	%	18.6	19.3
RBC	10^{12}cells/L	9.2	9.47
HGB	g/L	123	131
HCT	%	35.0	35.9
MCV	L	46.1	48
MCH	pg	15.8	15.9
MCHC	g/L	310	320
RDW	%	13.5	13.5
PLT	10^9cells/L	465	466
MPV	fL	5.8	6.0
PDW		14.9	14.8

c

Parameters	Control	DSF+COL
Glucose	5.6	6.2
Urea Nitrogen	8.10	6.29
Ca^{2+}	2.34	2.44
Creatinine	40	42
Albumin	30.3	27
Phosphorous	1.94	1.83
Glutamate transferase	46	44.8

Supplementary Figure 8 ***In vivo*** **toxicity evaluation of DSF-colistin combination**. CD-1 female mice (n = 6 biologically independent animals per group) were administered with vehicle or the colistin plus DSF (5 + 20 mg/kg) by intraperitoneal injection once daily for six days. The body weight **(a)**, serum biochemical analysis **(b)**, and whole-blood cell analysis **(c)** were measured. The data were presented as the mean of six biological samples. White blood cell (WBC), lymphocyte (LYM), monocyte (MON), neutrophils (NEU), red blood cell (RBC), hemoglobin (HGB), hematocrit (HCT = RBC%), the mean corpuscular volume (MCV, average volume of red cells), mean corpuscular hemoglobin (MCH), platelet count (PLT), and mean corpuscular hemoglobin concentration (MCHC, the average amount of hemoglobin inside a single red blood cell).

Tables

Supplementary Table 1 Antimicrobial susceptibility test of bacterial strains used in this study.

Pathogens and genotypes	Source	COL	MEM	TIG
E. coli 25922	ATCC	0.5	0.03125	0.25
S. enterica 13076	ATCC	0.125	0.0625	0.125
E. coli DH5α (PUC19)	In this study	<0.125	<0.125	1
E. coli B2 (*mcr-1* + $bla_{\mathrm{NDM-5}}$)	In this study	8	32	2
E. coli DH5α (PUC19-*mcr-1*)	In this study	2	32	1
E. coli G92 (*mcr-1*)	In this study	4	<0.125	4
E. coli CP131 (*mcr-3*)	In this study	2	<0.125	2
K. pneumoniae D120 (*mcr-8*)	In this study	4	<0.0625	0.25
E. coli C3 ($bla_{\mathrm{NDM-1}}$)	In this study	<0.125	8	2
E. coli G6 ($bla_{\mathrm{NDM-5}}$)	In this study	0.5	62	2
S. enteritidis H8 ($bla_{\mathrm{NDM-1}}$)	In this study	0.25	16	0.125

ATCC, American Type Culture Collection; MEM, meropenem; COL, colistin; TIG, tigecycline.

Supplementary Table 2 Synergistic activity of DSF in combination with different classes of antibiotic against *E. coli* B2.

Targets	Antibiotics	MIC[a] (μg/mL)	MIC[b] (μg/mL)	Potentiation (fold)[c]
DNA synthesis	Ciprofloxacin	32	32	–
Cell wall	Vancomycin	64	64	–
RNA synthesis	Rifampicin	128	128	–
Protein	Tigecycline	0.5	0.5	–
	Doxycycline	32	16	2
Cell wall	Meropenem	32	2	16
Cell membrane	Colistin	8	0.25	32
	+ 10% serum	8	0.5	16
	+ 10% DMEM	8	0.5	16
	+ 0.1 mM EDTA	2	0.0156	128
	+ 10 mM Mg^{2+}	16	8	2
	+ 10 mM Ca^{2+}	16	2	8
	+ 10 mM Na^{+}	8	0.5	16
	+ 10 mM K^{+}	8	0.5	16
	+ 10 mM NAC	32	16	2

[a/b]MICs of antibiotics in the absence or presence of sub-MIC of DSF.

[c]Degree of antibiotics potentiation in the presence of sub-MIC of DSF.

–, none of potentiation activity.

Supplementary Table 3 Potentiation of DSF derivatives with antibiotic against *E. coli* B2.

Derivatives	Antibiotics	MIC[a] (μg/mL)	MIC[a] (μg/mL)	Potentiation (fold)[c]
DDC	Ciprofloxacin	32	32	–
	Vancomycin	64	64	–
	Tigecycline	0.5	0.125	4
	Doxycycline	32	8	4
	Meropenem	32	2	16
	Colistin	8	0.5	16
DMDC	Ciprofloxacin	32	32	–
	Vancomycin	64	64	–
	Tigecycline	0.5	0.5	–
	Doxycycline	32	16	2
	Meropenem	32	4	8
	Colistin	8	0.5	16

[a/b]MICs of antibiotics in the absence or presence of sub-MIC of DSF derivatives.

[c]Degree of antibiotics potentiation in the presence of sub-MIC of DSF derivatives.

–, none of potentiation activity.

Supplementary Table 4 Synergistic activity of DSF, DDC and DMDC with meropenem against NDM-negative or -positive Gram-negative pathogens.

Compounds	Pathogens and genotypes	MIC[a] (μg/mL)	MIC[b] (μg/mL)	Potentiation (fold)[c]
DSF	*E. coli* ATCC 25922	0.031	0.031	–
	S. enterica ATCC 13076	0.063	0.063	–
	E. coli C3 (*bla*$_{\text{NDM-1}}$)	16	2	8
	E. coli G6 (*bla*$_{\text{NDM-5}}$)	32	2	16
DDC	*E. coli* ATCC 25922	0.031	0.031	–
	S. enterica ATCC 13076	0.063	0.063	–
	E. coli C3 (*bla*$_{\text{NDM-1}}$)	16	1	16
	E. coli G6 (*bla*$_{\text{NDM-5}}$)	32	4	8
DMDC	*E. coli* ATCC 25922	0.031	0.031	–
	S. enterica ATCC 13076	0.063	0.063	–
	E. coli C3 (*bla*$_{\text{NDM-1}}$)	16	2	8
	E. coli G6 (*bla*$_{\text{NDM-5}}$)	32	4	8

[a/b]MICs of meropenem in the absence or presence of sub-MIC of DSF and its derivatives.

[c]Degree of meropenem potentiation in the presence of sub-MIC of DSF and its derivatives.

Supplementary Table 5 Broad-spectrum synergistic activity of DSF, DDC and DMDC with colistin against MCR-negative or -positive Gram-negative pathogens.

Compounds	Pathogens and genotypes	MIC[a] (μg/mL)	MIC[b] (μg/mL)	Potentiation (fold)[c]
DSF	*E. coli* DH5α (PUC19)	0.25	0.063	4
	E. coli ATCC 25922	0.5	0.063	8
	S. enterica ATCC 13076	0.125	0.0078	16
	E. coli DH5α (PUC19-*mcr-1*)	2	0.125	16
	E. coli G92 (*mcr-1*)	4	0.063	64
	E. coli CP131 (*mcr-3*)	2	0.063	32
	K. pneumoniae D120 (*mcr-8*)	4	0.063	64
DDC	*E. coli* ATCC 25922	0.5	0.031	16
	S. enterica ATCC 13076	0.25	0.0156	16
	E. coli G92 (*mcr-1*)	4	0.125	32
	K. pneumoniae D120 (*mcr-8*)	4	0.125	32
DMDC	*E. coli* ATCC 25922	0.5	0.031	16
	S. enterica ATCC 13076	0.25	0.031	8
	E. coli G92 (*mcr-1*)	4	0.125	32
	K. pneumoniae D120 (*mcr-8*)	4	0.125	32

[a/b]MICs of colistin in the absence or presence of sub-MIC of DSF and its derivatives.

[c]Degree of colistin potentiation in the presence of sub-MIC of DSF and its derivatives.

Supplementary Table 6 Primers for RT-qPCR analysis in this study.

Genes	Primers (5'-3')
rpsJ	ACTGTTCTGATCTCCCCGCA
	TGTCAACCAGACGCAAGTGA
rpsY	TGACCATCGTTGTTGACGGTA
	CGAACGAAGTCGATGTGCTG
rpmE	CCCGTTCTTCACTGGCAAAC
	CCGGGATGTTGAAACGCTTG
appB	CGACGGATTTGACATGGGGA
	AAATAATGCCCCACCAGCGA
atpG	GCCGCTTCCAAAATGCGTAA
	TTTAACGTCGCGGTCTTCCA
frdB	GAGCTGTACTTTCGTGGGCT
	TTAGCGTGGTTTCAGGGTCG
carA	ATTCGGCATCTGTCTCGGTC
	GGTTCTGGGCGGTGATCATT
pyrB	CAACCAACATCCGACGCAAA
	GAGTCAGGGAGTGAACGGTG
upp	GGGTAGCCTGCTGACTTACG
	CCATCATACCAAGACCCGCA
mcr-1	AAAGACGCGGTACAAGCAAC
	GCTGAACATACACGGCACAG
16S rRNA	CCTACGGGAGGCAGCAG
	ATTACCGCGGCTGCTGG

Supplementary Table 7 Energy of conformer values of docking results, related to Figure 2e, Supplementary Figure 6.

	Modes	DSF-NDM-1	DDC-NDM-1	DMDC-NDM-1
Affinity (kcal/mol)	1	-5.14	-3.26	-3.45
	2	-5.08	-3.25	-3.32
	3	-4.99	-3.23	-3.29
	4	-4.82	-3.21	-3.20
	5	-4.70	-3.20	-3.16
	6	-4.69	-3.19	-3.14
	7	-4.66	-3.14	-3.13
	8	-4.63	-3.13	-3.11
	9	-4.62	-3.12	-3.07
	10	-4.53	-3.09	-3.05

Supplementary Table 8 Energy of conformer values of MCR docking results, related to Figure 4b, Supplementary Figure 7.

	Modes	DSF-MCR-1	DDC-MCR-1	DMDC-MCR-1
Affinity (kcal/mol)	1	-3.75	-2.54	-2.69
	2	-3.70	-2.53	-2.68
	3	-3.65	-2.52	-2.66
	4	-3.54	-2.51	-2.66
	5	-3.51	-2.43	-2.54
	6	-3.46	-2.37	-2.45
	7	-3.43	-2.34	-2.42
	8	-3.43	-2.27	-2.39
	9	-3.37	-2.16	-2.38
	10	-3.27	-2.11	-2.33

Supplementary Table 9 MIC values of MEM and COL against NDM-positive *E. coli* C3 or MCR-positive *E. coli* G92 without/with DSF, respectively.

Passages	MICs of MEM (μg/mL)		MICs of COL (μg/mL)	
	- DSF	+ DSF	- DSF	+ DSF
0	8	8	4	4
2	8	8	4	4
4	8	8	4	4
6	8	8	8	4
8	16	8	8	4
10	16	8	8	4
12	16	8	16	4
14	16	8	16	4
16	16	8	16	4
18	32	8	32	4
20	32	8	32	4
22	64	8	32	4
24	64	8	32	4
