# Supplementary material for: Resensitizing multidrug-resistant Gram-negative bacteria to carbapenems and colistin using disulfiram
Source: Commun Biol. 2023 Aug 3;6:810. doi: 10.1038/s42003-023-05173-7 (PMC10400630; doi:10.1038/s42003-023-05173-7)
Supplement: Supplementary file 2 — Description of Additional Supplementary Files [file 42003_2023_5173_MOESM2_ESM.pdf]

Description of Additional Supplementary Files

File Name: Supplementary Data 1
Description: Source data for Figures 1-7.
